# Comprehensive datasets for RNA design, machine learning, and beyond

**DOI:** 10.1038/s41598-025-07041-2

**Published:** 2025-07-01

**Authors:** Jan Badura, Agnieszka Rybarczyk, Tomasz Zok

**Affiliations:** 1 Institute of Computing Science, Poznan University of Technology, 60-965 Poznan, Poland; 2 Institute of Bioorganic Chemistry, Polish Academy of Sciences, 61-704 Poznan, Poland

**Keywords:** RNA design, Dataset, Machine learning, RNA structure, Multiloop, n-way junction, Computational biology and bioinformatics, Data processing

## Abstract

RNA molecules are essential in regulating biological processes such as gene expression, cellular differentiation, and development. Accurately predicting RNA secondary structures and designing sequences that fold into specific configurations remain significant challenges in computational biology, with far-reaching implications for medicine, synthetic biology, and biotechnology. While machine learning methodologies have been proposed to enhance prediction capabilities, they require high-quality training data. The lack of standardized benchmark datasets further hinders the development and evaluation of these tools. To address this, we created a comprehensive dataset of over 320 thousand instances from experimentally validated sources to establish a new community-wide benchmark for RNA design and modeling algorithms. Our dataset comprises numerous challenging structures for which state-of-the-art RNA inverse folders provide results of varying accuracy. We demonstrated the potential of the dataset by testing it with several popular open-source RNA design algorithms. Furthermore, we illustrated how our dataset can be used to train machine learning models that consider both RNA sequence and structure, potentially advancing RNA design and prediction capabilities.

## Introduction

RNA molecules play a crucial role in living organisms, regulating a variety of biological processes such as gene expression, cellular differentiation, and development^1,2^. These diverse functions rely on the capacity of single-stranded RNA molecules to adopt a particular structure^3^. Initially, the RNA molecule folds into secondary structure through canonical Watson–Crick and wobble base pairing, which then guides the formation of the three-dimensional shape, known as the tertiary structure^4^. Therefore, accurately predicting RNA secondary structure and designing RNA sequences that fold into specific structures are major challenges in computational biology. These tasks hold significant implications for medicine, synthetic biology, and biotechnology^5–8^.

Since the 1970s, the prediction of RNA secondary structure has primarily been studied and approached through computational methods^9–12^. Most of these methods employ dynamic programming and thermodynamic calculations to identify the structure with minimum free energy (MFE), based on the principle that RNA molecules, like proteins, exist in energetically stable states^13^. Over the years, numerous software applications have been developed that incorporate these methods^14–18^. However, in the last decade, improvements in prediction accuracy and calculation speed have remained limited^19^. To address this issue, methodologies based on machine learning (ML), which have achieved significant success for the first time in protein structure prediction with AlphaFold^20,21^, have been proposed to improve the prediction of the secondary structure of RNA^17,19^. Unlike classical methods that rely heavily on thermodynamic mechanics and labor-intensive experimental data, ML approaches make fewer assumptions, making them better suited for detecting complex foldings, such as non-canonical base pairing or previously unrecognized base pairing constraints^22^.

Generally, they can be classified into three categories: ML-based scoring schemes, ML-based preprocessing and postprocessing, and ML-based predictions^22^. All ML-based methods in these three categories train their models using supervised learning. Within this framework, there are numerous proposals, each differing in architectural design, input-output of the model, training data, and optimization algorithms, for example, SPOT-RNA^23^, SPOT-RNA2^24^, MXFold2^25^, UFold^26^, Contextfold^27^ and CONTRAfold^28^. However, their prediction accuracy still leaves much room for improvement, mainly due to the ongoing challenge of collecting sufficient, representative, and high-quality training data, which limits the potential of ML methods^22,29^.

A reliable solution to the folding of the RNA structure is essential to address the significant challenge of the inverse folding of RNA, which involves designing RNA sequences that fold into a desired secondary or tertiary structure to perform a specific function^30,31^. This problem can be defined as the inverse problem of RNA folding.

Recent advances in deep learning, along with the increasing availability of biomolecular structural data, have driven the development of algorithms specifically designed to tackle the RNA 3D inverse folding task^32^. However, most existing methods still learn only limited 3D structural features from experimentally determined or predicted 3D structure datasets^32–34^, and only a few recent studies have begun to address this problem directly^32,35–37^. Among these, RiboDiffusion is a deep generative diffusion model for RNA inverse folding that learns RNA sequence distribution conditioned on fixed 3D backbone structures, combining graph neural networks with a Transformer-based architecture to capture both geometric and sequence dependencies^32^. RIdiffusion, in turn, extends this approach by introducing a hyperbolic denoising diffusion generative model for 3D RNA inverse folding, parameterized by hyperbolic equivariant graph neural networks (HEGNNs), enabling efficient modeling of hierarchical structural variations. This approach enhances representational efficiency and improves model performance, particularly in low-data settings^37^. Finally, gRNAde is a geometric deep learning framework for RNA 3D inverse folding, analogous to ProteinMPNN^38^ in the context of protein design. It is designed to handle single-state and multi-state fixed-backbone sequence design, enabling it to account for the conformational flexibility of RNA^35,36^.

While these models demonstrate promising results in capturing the geometric and topological complexities of RNA tertiary structures, they face significant limitations. One of the key challenges is the shortage of experimentally determined high-resolution RNA 3D structures, which severely limits the amount of training data available, especially compared to proteins, for which structural data are far more abundant^29^. Furthermore, the inherent structural flexibility of RNA and the non-unique mapping between sequence and structure further complicate accurate modelling. Unlike proteins, which typically fold into a relatively stable conformation, RNA molecules are highly dynamic and can adopt multiple distinct structural states depending on environmental conditions, ligand interactions, or cellular context^37,39,40^.

In contrast, secondary structures are supported by large databases, are easier to predict reliably, and often sufficient for capturing the key functional motifs of RNA^30^. Thus, inverse RNA folding at the secondary structure level remains a powerful strategy, especially for designing functional elements such as RNA switches^41^. These synthetic regulatory elements respond to molecular signals by undergoing conformational changes, enabling precise control over gene expression. Their programmability, efficiency, and functional versatility make 2D-based inverse folding a practical and scalable solution in synthetic biology, particularly in applications such as therapeutics, diagnostics, and cellular engineering^30,42,43^. For these reasons, this work focuses in particular on 2D inverse folding models.

Since testing each sequence to see if its minimum free-energy structure matches the target is impractical because the number of sequences grows exponentially with the size of the structure, current inverse RNA folding algorithms employ a variety of heuristic methods rather than exploring the entire solution space^5,30^. For example, tools such as INFO-RNA^44^, Modena^45^, RNAinverse^46^, RNAsfbinv^47^, and DSS-Opt^48^ use local search methods, while DesiRNA^49^ and MCTS-RNA^50^ employ Monte Carlo algorithms. Furthermore, m2dRNAs^51^ uses multi-objective optimization, whereas RNARedPrint^52^ combines Boltzmann sampling with dynamic programming over tree decomposition to efficiently handle complex design targets. Others, such as RNAiFold^53^ and MoiRNAiFold^54^, are based on constraint programming, with MoiRNAiFold inheriting the design constraints and philosophy of RNAiFold while introducing new modeling concepts to enhance its efficiency. Finally, the deep reinforcement learning-based algorithm Meta-LEARNA^55,56^ provides a pretrained model with optimized parameters, obtained through pretraining on a large corpus of biologically relevant sequences, enabling efficient generalization across diverse RNA design tasks. However, as with ML-based methods for RNA secondary structure prediction, the absence of standardized benchmark datasets presents a significant challenge for the development and evaluation of tools in this field.

Currently, the only data set available and recognized by the scientific community for this purpose is EteRNA100, a collection of structures assembled manually by experts^57^. This set includes 100 distinct secondary structure design challenges, with lengths ranging from 12 to 400 nucleotides and an average length of 127 nucleotides. It includes a variety of structures, highlighting the challenges in the design of RNA and incorporating different combinations of secondary structure elements.

Unfortunately, the lack of a single common standard for evaluation protocols for the Eterna100 dataset makes it difficult to compare and assess different RNA design algorithms consistently. To address this issue, a new RNA benchmark library called RnaBench has recently been proposed, specifically designed for the development of deep learning algorithms^58^. It includes benchmarks for the modeling of RNA structures, homology-aware curated datasets, standardized evaluation protocols, novel performance measures and a visualization module. However, it focuses exclusively on tasks related to the prediction of RNA secondary structure and the design of RNA.

Although the Eterna100 and RnaBench benchmarks cover a wide spectrum of design features and difficulties, it should be noted that all the structures they contain are less than 500 nucleotides long^57,59^. Since the advancement of sequencing technologies has revolutionized transcriptome research, it has led to an increase in the length and complexity of RNA^60^. This, in turn, increased the number of asymmetric and symmetric components, heightening the challenge of designing sequences for these molecules. To further assess the capability of different RNA design methods in the design of long secondary structures, users need to independently choose and prepare extended test sets.

Thus, to address the need for a new community-wide standard benchmark specifically designed for RNA design and RNA modeling algorithms, we made use of experience in our previous resource^61^ and created a very large, comprehensive and general-purpose dataset of over 320 thousand secondary structures with lengths ranging from 5 to 3,538. Our focus was mainly on multi-branched loops, which are often challenging to predict accurately^62,63^. Consequently, this data set encompasses a diverse range of complex motifs, from internal loops to n-way junctions (loops with *n* outgoing helices, where $n\ge 3$), all extracted from RNA structures available in the RNAsolo^64^ and Rfam^60,65^ databases. We also tested this new data set using several popular and open-source RNA design algorithms, including RNAinverse, INFO-RNA, DSS-Opt, RNAsfbinv, RNARedPrint, Meta-LEARNA, and DesiRNA.

## Results

### Dataset content

We have compiled a comprehensive dataset featuring 4,921 loop motifs from the RNAsolo database. Most notably, 82.4% of these motifs are internal loops, each averaging about 67 nucleotides in length (counting the motif itself and the connecting stems). Following closely are 3-way and 4-way junctions, making up 9.49% and 6.38% of the dataset, with average lengths of 143 and 154 nucleotides respectively. The dataset also includes a single instances of loops with cardinalities as high as 8- and 10-way junctions and lengths extending to several thousand nucleotides. These extreme cases are likely outliers, possibly stemming from inherent uncertainties in the PDB structures and the annotating software that processes them. Detailed statistics can be found in Table 1.

**Table 1:** Statistics of loop motifs with connecting stems extracted from the RNAsolo database.

Type	Count	Percent	Length			
			Min	Max	Mean	Std. dev.
Internal loop	4055	82.4	8	3049	66.5	103.34
3-way junction	467	9.49	24	571	142.7	116.83
4-way junction	314	6.38	49	1099	154.24	177.06
5-way junction	68	1.38	70	1625	349.88	317.72
6-way junction	9	0.18	248	632	385.78	134.05
7-way junction	6	0.12	379	2927	1225.67	1304.73
8-way junction	1	0.02	3117	3117	3117.0	
10-way junction	1	0.02	3041	3041	3041.0	
Total	4921					

The dataset based on all Rfam sequences boasts an impressive 320 thousand loop motif instances. Analyzing post-processed data from the RNAfold pipeline reveals that, much like the RNAsolo dataset, internal loop motifs dominate, accounting for 85.29% of the total instances. Additionally, 3-way and 4-way junctions make up 9.18% and 3.99% of instances respectively.

The average lengths of these prevalent motifs in Rfam are approximately 69 nucleotides for internal loops, 128 nucleotides for 3-way junctions, and 155 nucleotides for 4-way junctions. Similar to RNAsolo, the dataset includes some outliers such as 9-, 10-, and 12-way junctions and sequences extending several thousand nucleotides. These extreme cases likely arise from data uncertainties, as some RNA families have short alignments and weak covariance signals, leading to significantly underfolded consensus 2D structures.

We further investigated the origins of the most extreme outliers in our data. The instances of 10-way junctions are derived from the RNAIII family (Rfam ID: RF00503). While the current consensus structure for this family features a 9-way junction with flexible regions, we found that applying these constraints to two specific sequences within a small alignment (consisting of 23 sequences) resulted in the 10-way junctions. It is important to note that this alignment has very weak statistical support, with only 4 out of 132 base pairs being statistically significant. This limited support reinforces our classification of this large junction as an outlier, likely arising from data limitations. Additionally, the dual biological role of RNAIII sequences—regulating processes and coding for a small protein in *Staphylococcus aureus*^66^—makes the formation of such a high-order junction less probable than that of multiple distinct stem-loops, which are typical of transcripts. This further justifies treating it as an outlier.

In contrast, all identified 12-way junctions trace back to Archaeal large subunit ribosomal RNA (LSU rRNA, Rfam ID: RF02540). This family has an extensive alignment of 3,046 sequences, with strong statistical support for many base pairs (452 out of 786). Its consensus secondary structure contains a central high-order junction, which leads to the formation of 12-way instances when applied to 91 sequences. Importantly, the 3D structure of Archaeal LSU rRNA has been experimentally determined (e.g., PDB ID: 6TH6 for *T. kodakarensis* 70S ribosome^67^). Our analysis of this structure confirmed the presence of complex multi-junctions, with up to 7-way junctions observed when excluding pseudoknotted stems. Including pseudoknotted stems—a debatable practice we avoided in our primary analysis – reveals even higher-order arrangements (e.g., 11- and 17-way junctions). Therefore, while the specific 12-way junction derived from the Rfam pipeline might still represent an outlier or artifact, it reflects genuine biological complexity involving high-order multi-junctions, likely formed by combinations of lower-order junctions (such as 7-way and 5-way). This investigation highlights the inclusion of structures in the dataset that push the boundaries of current modeling capabilities, stemming from data limitations and true biological complexity.

For a detailed breakdown, refer to Table 2, where all specifics about this comprehensive dataset are documented.

The choice between using data from our dataset’s RNAsolo or Rfam components depends on the specific research objective. The RNAsolo dataset (Table 1), which is curated from non-redundant, experimentally determined 3D structures, offers high-confidence 2D structure annotations based on empirical evidence. Although RNAsolo is limited in size, it is particularly well-suited for situations that require the highest confidence in the ground truth structure, such as benchmarking structure prediction methods that depend on accuracy compared to experimentally validated structures.

**Table 2:** Statistics of loop motifs with connecting stems extracted from the Rfam database (with RNAfold post-processing).

Type	Count	Percent	Length			
			Min	Max	Mean	Std. dev.
Internal loop	273350	85.29	5	3078	68.7	80.82
3-way junction	29410	9.18	26	1194	128.04	111.07
4-way junction	12779	3.99	43	1004	154.65	126.09
5-way junction	3512	1.1	66	2040	356.07	280.92
6-way junction	802	0.25	101	1275	304.18	100.26
7-way junction	348	0.11	198	3457	670.26	764.68
8-way junction	174	0.05	185	3446	1385.71	1257.26
9-way junction	8	0.002	220	375	331.25	50.3
10-way junction	2	0.0006	279	337	308.0	41.01
12-way junction	91	0.03	2850	3538	2976.62	95.5
Total	320476					

On the other hand, the Rfam dataset (Table 2) is based on seed alignments and consensus secondary structures, providing a much larger scope and greater diversity across various RNA families. However, this extensive coverage has the disadvantage of being highly unbalanced, with varying levels of annotation confidence. Some families exhibit strong covariance signals and robust annotations. In contrast, others may not have enough support. Therefore, Rfam is better suited for large-scale analyses, broader family coverage, or training data-intensive models, provided that users know and account for the inherent imbalance and differing levels of annotation confidence among families.

### Comparison with existing RNA design benchmarks: Eterna100 and RnaBench

Eterna100 stands out as a manually curated set of 100 synthetic RNA design challenges created by the Eterna online community^57^. These puzzles were specifically chosen to expose the limitations of existing RNA design methods by incorporating motifs that tend to be energetically unstable across sequence space, thus increasing the likelihood of competing suboptimal structures. As a result, Eterna100 has played a key role in identifying structural features that consistently lead to failure in both algorithmic and human-guided RNA design^57^.

The inverse RNA folding dataset within the RnaBench library, on the other hand, is a compilation of datasets proposed by various authors, primarily used to evaluate the performance of their own RNA inverse folding methods, and made available through their public repositories. It includes several test sets based on a limited number of selected Rfam entries denoted as: Rfam Taneda dataset^45^, Rfam Kleinkauf dataset^68^, Rfam LEARN dataset^55^, as well as a test set based on RNA-Strand (RNA-Strand Kleinkauf dataset)^68^ and the Eterna100 benchmark^57^. It also includes a set of pseudoknot-containing samples taken from the Chen dataset^69^, which was constructed based on examples from ArchiveII^70^.

A comparative overview of these datasets, including coverage (in terms of number of samples) and sequence length diversity, is provided in Table 3. Furthermore, since Eterna100 is included in the RnaBench dataset, we performed a detailed comparison between the content of the RnaBench dataset and the dataset proposed in this study. The results of this comparison are presented in the Table 4. In particular, only 64 samples are shared between the two datasets, which clearly demonstrates that our dataset complements the existing resources.

**Table 3:** Statistics of benchmark datasets with sequence length distribution.

Benchmark	Nr of samples	Length 1–500	${\textrm{Length }}> {\textrm{500}}$
Eterna	100	100	0
RnaBench (Inverse RNA Folding Dataset)	68553	68553	0
Our dataset (loop motifs with connecting stems extracted from the RNAsolo database)	4921	4840	81
Our dataset (loop motifs with connecting stems extracted from the Rfam database)	320476	316832	3644

**Table 4:** A detailed comparison of the contents of the RnaBench dataset (Inverse RNA Folding Dataset) with our dataset. If the number of different samples for a given ID is greater than one, the number is provided in parentheses next to the ID.

Compared datasets	RFAM/PDB id	Avg. structure length (std. dev.)	No. of shared samples
RnaBench vs Our Dataset (samples extracted from the Rfam database)	RF00001, RF00005 (2), RF00007, RF00014, RF00019, RF00020, RF00021, RF00026, RF00029, RF00037, RF00043, RF00047, RF00053, RF00056, RF00090, RF00103, RF00167, RF00231, RF00237, RF00322, RF00400, RF00404, RF00406, RF00413, RF00422, RF00424, RF00446, RF00451, RF00545, RF00553, RF00565, RF00568, RF00582, RF00617, RF00641, RF00657, RF00667, RF00679, RF00906, RF00951, RF01225, RF01234, RF01241, RF01418, RF01751, RF01782, RF01797, RF01844, RF02030, RF02097, RF02635, RF02689, RF02723, RF02736, RF02737, RF02741, RF02742, RF02749, RF02755	87.4 (72.3)	60
RnaBench vs Our Dataset (samples extracted from the RNAsolo database)	1JOX_1_A, 1R2P_9_A, 1U3K_7_A, 7UW1_1_B	51.75 (36.47)	4

It is also worth noting that, while RnaBench is based on secondary data sources, our dataset relies on primary data sources, offering a more direct and up-to-date representation of RNA structures. It offers a comprehensive representation of the RNAsolo and Rfam database and significantly broadens the range of structure lengths, covering both short and long ones, including thousands of motifs exceeding 500 nucleotides.

We recognize that traditional inverse folding algorithms often face significant computational challenges as the sequence length increases, which can limit their practical application. However, the field is advancing, with recent findings highlighting new approaches capable of handling longer sequences^71^. We believe it is essential for a benchmark dataset to be forward-looking. Therefore, including very long sequences is necessary to test current methods’ limits and create a relevant and challenging framework for future algorithms designed for such sequences. This approach ensures that the benchmark remains valuable as RNA design capabilities evolve.

In addition, our dataset addresses the need for more complex RNA structures, particularly those derived from high-resolution experimental data. It includes over 320,000 loop motifs extracted from Rfam and RNAsolo, encompassing internal loops, 3-way, 4-way, and higher-order junctions.

### Recognizing glutamine riboswitch

To showcase the potential of our dataset in machine learning pipelines, we embarked on training a binary classification model to identify glutamine riboswitches (RFAM id: RF01739) based on their secondary structure. We selected glutamine riboswitches for this initial demonstration because of their unique structural junction, which holds considerable biological significance. This characteristic is subject to evolutionary pressure, leading to its conservation across aligned sequences. As a result, it serves as a suitable and straightforward example for a classification task based on junction features. These riboswitches are distinguished by a characteristic 3-way junction with an E-loop motif^72–74^, which we hypothesize can be differentiated from other RNAs featuring 3-way junctions^74^.

**Fig. 1:**
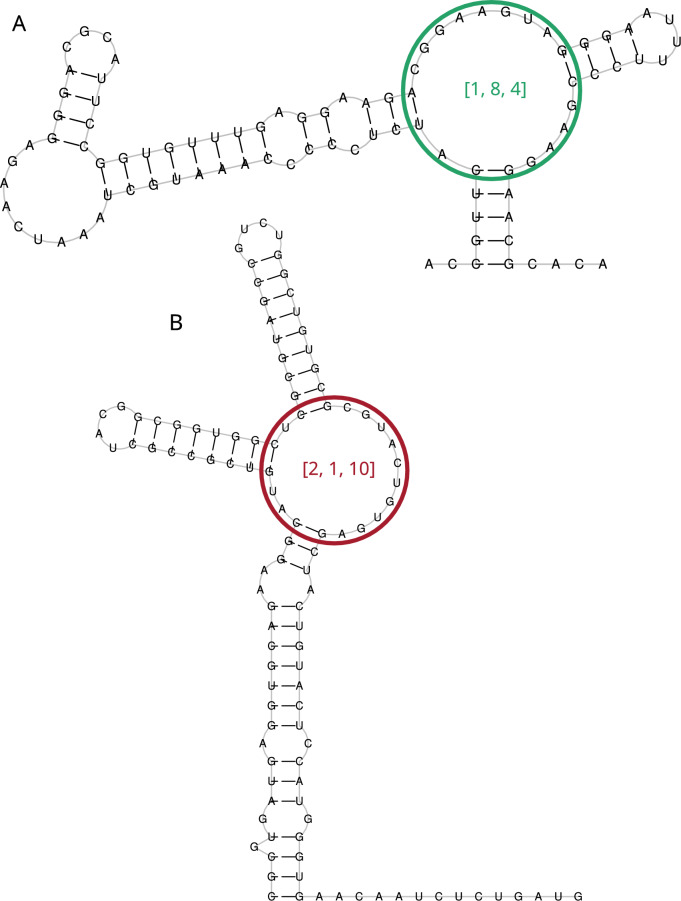
Example features for machine learning model—counts of unpaired residues in a multiloop. (**A**) Glutamine riboswitch from Planktothrix agardhii (a positive example in our training set). (**B**) Small nucleolar RNA TBR2 from Trypanosoma brucei (a negative example).

Our first task was data preparation. From the Rfam-derived dataset we report in this paper, we isolated entries containing 3-way junctions, representing each as a vector of four values: three integers denoting the counts of unpaired residues in the three strands of the multiloop and a decision variable (indicating whether it is a glutamine riboswitch or not) (see Fig. 1). This yielded 29,410 vectors, with 937 of them being glutamine riboswitches.

We proceeded to train three classifiers: k-Nearest Neighbours (k = 3), a Decision Tree, and Naive Bayes. These models were evaluated based on the F1-score macro average, particularly vital for such highly imbalanced dataset. Employing a stratified 5-fold cross-validation technique, we observed the performance of the models across different subsets.

The Decision Tree classifier outperformed the others, achieving the highest F1-score in each fold, with an impressive average F1-score of 0.998. The Naive Bayes classifier closely followed, reaching an average F1-score of 0.995. In contrast, the kNN classifier had the lowest performance in the last two folds, resulting in an average F1-score of 0.988. Detailed results are in Table 5.

**Table 5:** F1-scores in stratified 5-fold cross-validation in the problem of recognizing glutamine riboswitch.

Fold	kNN (k = 3)	Decision tree	Naive Bayes
1	0.997	0.999	0.997
2	0.994	0.996	0.992
3	0.996	0.996	0.993
4	0.979	1.0	0.996
5	0.975	0.999	0.997
Min	0.975	0.996	0.992
Max	0.997	1.0	0.997
Median	0.994	0.999	0.996
Mean	0.988	0.998	0.995

We utilized a straightforward model and implemented basic classical machine learning methods to demonstrate that, thanks to the dataset presented in this manuscript, it is indeed possible to train a classifier to recognize an Rfam family based on its characteristic features. However, the simplicity of the model and classifiers used does not impose any limitations. We envision that others could investigate more sophisticated hypotheses and create advanced models with improved features by leveraging our robust dataset.

Although the binary classification example illustrates the dataset’s utility, employing only basic features, such as unpaired residue counts, and standard machine learning algorithms like KNN, decision trees, and Naive Bayes limits the exploration of the dataset’s potential for the advanced applications outlined in the title and introduction. A comprehensive investigation using more sophisticated machine learning workflows warrants a separate study and falls outside the scope of this article. Nevertheless, our discussion section addresses this potential further.

### Evaluation and comparison of RNA design algorithms’ performance

The proposed datasets were used to evaluate and compare the performance of various RNA design tools, such as RNAinverse, INFO-RNA, DSS-Opt, RNAsfbinv, RNARedPrint, Meta-LEARNA, and DesiRNA. The first test was performed using a dataset derived from the RNAsolo database. For the second example, given the enormous size of the dataset derived from the Rfam database, we decided to demonstrate its capabilities using three specific families, each featuring distinct structural motifs that pose challenges for modeling: the glutamine riboswitch (RFAM id: RF01739), which features a characteristic three-way junction with an E-loop motif, the twister sister ribozyme (RFAM id: RF02681), and nuclear ribonuclease P (RFAM id: RF00009). The second family, the self-cleaving twister sister ribozyme, adopts either a three-way or four-way junctional fold, linked by internal and terminal loops. These loops, which contain conserved residues, closely resemble those found in the twister ribozyme^75,76^. The last family, characterized by the most complex secondary structure, includes nuclear ribonuclease P (RNase P), a ubiquitous endoribonuclease responsible for cleaving precursor tRNA transcripts to produce their mature $5^\prime$ termini. While the archaeal and eukaryotic holoenzymes contain significantly more protein components compared to their bacterial counterparts, the RNA core structure is conserved across RNase P RNAs from different species. This core consists of five critical regions with conserved nucleotides and several helices (P1, P2, P3, P4, and P10/P11) occupying similar positions in the RNA structures. Despite this structural conservation, there is notable sequence variation, particularly among eukaryotic RNAs^77,78^.

Presenting detailed analyses of the selected Rfam families is particularly informative. The performance evaluation showed inconsistent results across different runs or tools for these cases. This variability highlights two critical points: first, the sensitivity of current inverse folding algorithms to the nuances of input data, which underscores the challenges posed by these complex structures, and second, the potential value of using well-defined subsets derived from our comprehensive dataset for rigorously benchmarking, validating, or fine-tuning newly developed methods.

#### Benchmarking test case using a dataset of loop motifs derived from the RNAsolo database

Due to the varying accuracy levels of different RNA design tools across cases of different lengths, an analysis was performed on the common instances addressed by all tools (see Table 6 for more details). The performance of these tools was then compared using three metrics, RNAdistance, RNApdist and F1-score.

**Table 6:** RNA design benchmark results for the whole RNAsolo dataset (best values in bold).

RNA design algorithm	No of solved cases	Average computing time (s)	Normalized RNAdistance	RNApdist	F1-score
Results for 4921 instances					
RNAinverse	4452	2.32	0.12	13.08	0.92
RNAsfbinv	4161	7.74	0.23	13.09	0.65
INFO-RNA	4163	** 1.44**	0.20	14.19	0.76
DSS-Opt	**4913**	3.69	0.13	26.09	0.89
DesiRNA	4638	319.72	0.09	15.81	0.92
RNARedPrint	4393	11.11	0.29	22.78	0.77
Meta-LEARNA	2748	5.26	**0.06**	**9.38**	**0.94**
Results for 2575 instances successfully solved by each algorithm					
RNAinverse	2575	0.37	0.06	9.36	0.97
RNAsfbinv	2575	5.47	0.10	10.34	0.85
INFO-RNA	2575	**0.06**	0.13	9.14	0.84
DSS-Opt	2575	2.16	0.05	8.84	0.96
DesiRNA	2575	305.21	**0.01**	**8.45**	**0.99**
Meta-LEARNA	2575	4.99	0.06	8.67	0.94
RNARedPrint	2575	7.85	0.14	9.45	0.90

**Fig. 2:**
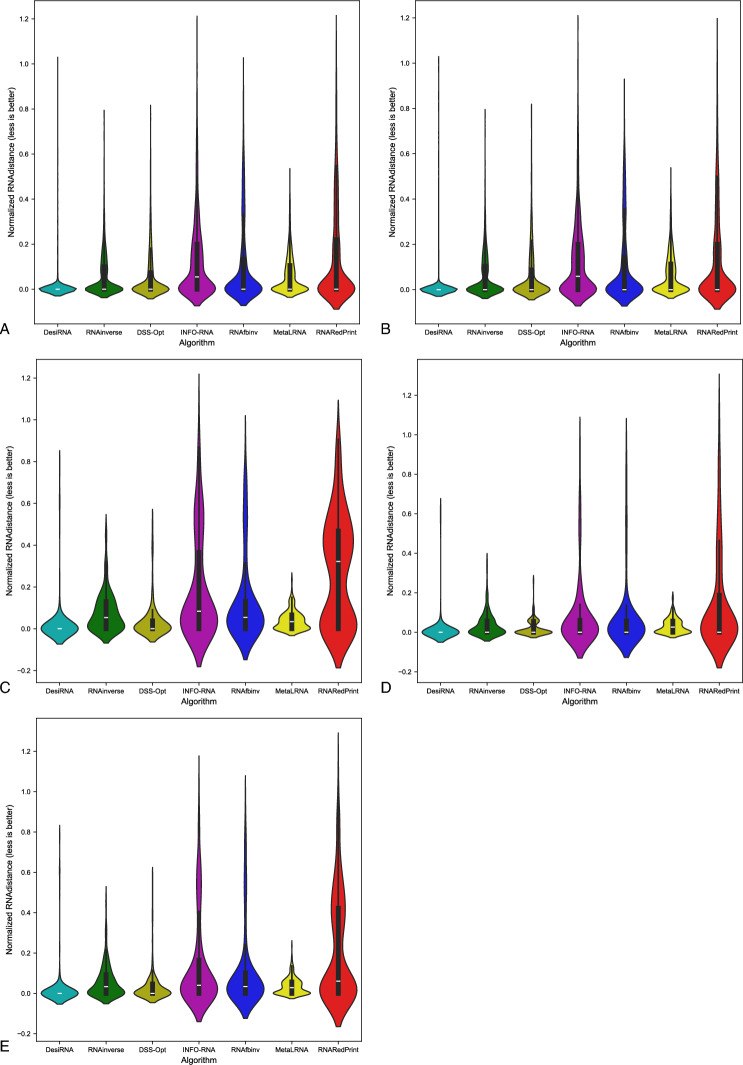
RNA design tools’ performance on RNAsolo dataset using normalized RNAdistance for benchmarking. (**A**) The entire set. (**B**) The subset that contains internal loops. (**C**) The subset that contains 3-way junctions. (**D**) The subset that contains 4-way junctions. (**E**) The subset that contains other higher-cardinality junctions. The Meta-LEARNA algorithm is labeled as MetaLRNA in the figure for short.

**Fig. 3:**
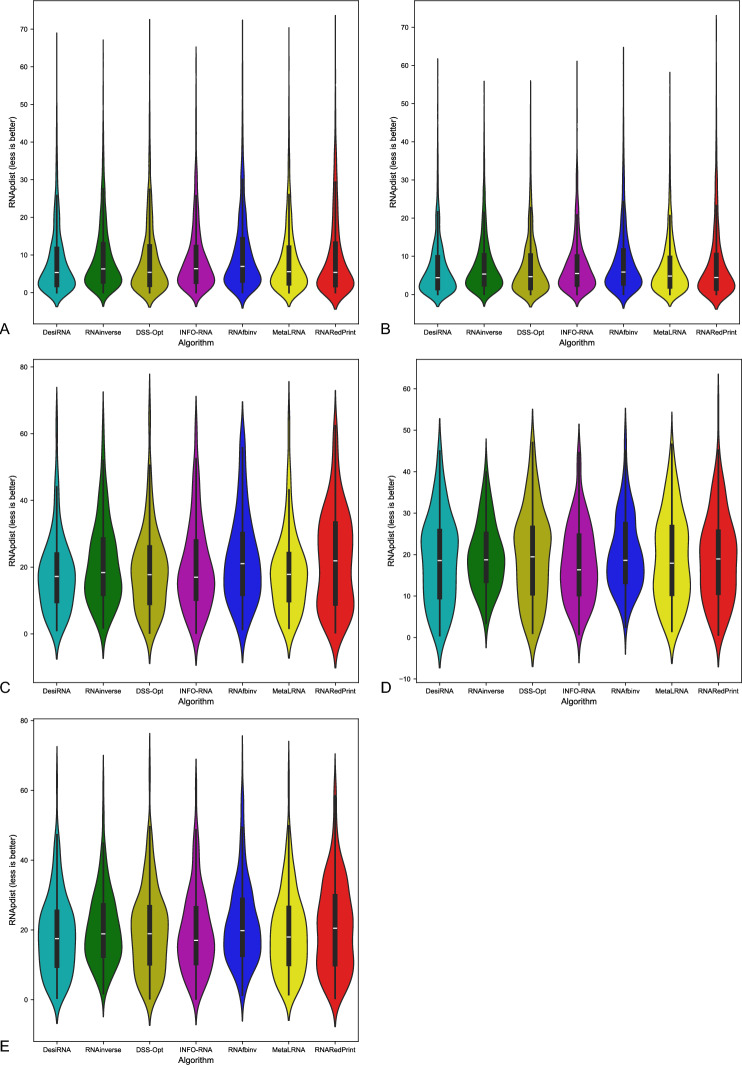
RNA design tools’ performance on RNAsolo dataset using RNApdist for benchmarking. (**A**) The entire set. (**B**) The subset that contains internal loops. (**C**) The subset that contains 3-way junctions. (**D**) The subset that contains 4-way junctions. (**E**) The subset that contains other higher-cardinality junctions. The Meta-LEARNA algorithm is labeled as MetaLRNA in the figure for short.

**Fig. 4:**
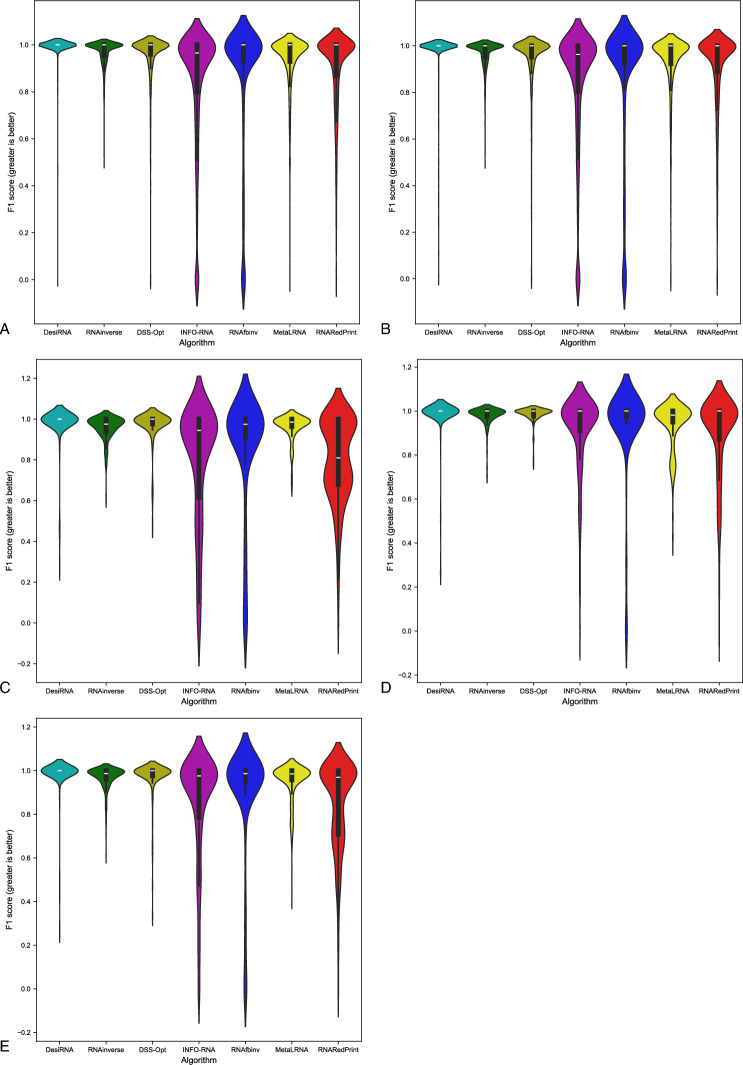
RNA design tools’ performance on RNAsolo dataset using F1-score for benchmarking. (**A**) The entire set. (**B**) The subset that contains internal loops. (**C**) The subset that contains 3-way junctions. (**D**) The subset that contains 4-way junctions. (**E**) The subset that contains other higher-cardinality junctions. The Meta-LEARNA algorithm is labeled as MetaLRNA in the figure for short.

Since our set encompasses a diverse range of difficult-to-design multiloop motifs, we have separately evaluated the performance of the RNA design algorithms on the following subsets of our dataset: internal loops and other higher-cardinality junctions, from which we further distinguished two additional subsets: 3-way junctions and 4-way junctions. The results are presented in Table 7 as well as Figs. 2, 3 and 4.

**Table 7:** RNA design benchmark results for the RNAsolo dataset divided by motif type (best values in bold).

RNA design algorithm	Average computing time (s)	Normalized RNAdistance	RNApdist	F1-score
Results for 2248 instances of internal loop motifs successfully solved by each algorithm				
RNAinverse	0.24	0.06	7.73	0.97
RNAsfbinv	4.17	0.10	8.68	0.85
INFO-RNA	**0.05**	0.12	7.65	0.84
RNARedPrint	7.69	0.13	7.75	0.91
DSS-Opt	2.13	0.06	7.24	0.96
DesiRNA	293.50	**0.01**	**6.95**	**0.99**
Meta-LEARNA	4.86	0.07	7.13	0.94
	**8.13**			
Results for 327 instances of higher-cardinality junction motifs successfully solved by each algorithm				
RNAinverse	1.31	0.06	20.59	0.97
RNAsfbinv	14.42	0.11	21.72	0.88
INFO-RNA	**0.16**	0.14	19.43	0.85
RNARedPrint	8.92	0.22	21.08	0.84
DSS-Opt	2.33	0.03	19.82	0.98
DesiRNA	385.78	**0.02**	**18.75**	**0.99**
Meta-LEARNA	5.88	0.04	19.27	0.95
Results for 160 instances of 3-way junction motifs successfully solved by each algorithm				
RNAinverse	1.91	0.08	21.21	0.96
RNAsfbinv	19.72	0.14	22.60	0.84
INFO-RNA	**0.17**	0.20	20.23	0.78
RNARedPrint	9.37	0.28	22.54	0.80
DSS-Opt	2.91	0.04	19.33	0.97
DesiRNA	384.37	**0.02**	**18.12**	**0.98**
Meta-LEARNA	6.65	0.04	18.93	0.96
Results for 154 instances of 4-way junction motifs successfully solved by each algorithm				
RNAinverse	0.74	0.04	19.70	0.98
RNAsfbinv	8.73	0.07	20.35	0.92
INFO-RNA	**0.13**	0.09	**18.14**	0.91
RNARedPrint	8.44	0.15	19.12	0.90
DSS-Opt	1.76	0.02	19.76	**0.99**
DesiRNA	386.64	**0.01**	18.81	**0.99**
Meta-LEARNA	4.78	0.03	19.13	0.94

The analysis of results for F1-score, RNApdist, and normalized RNAdistance metrics across seven algorithms (RNAinverse, INFO-RNA, DSS-Opt, RNAsfbinv, RNARedPrint, DesiRNA, Meta-LEARNA) on the entire dataset and its subsets reveals distinct patterns. DesiRNA consistently produces sequences with structures closely aligned to the target, as reflected by the lowest median RNAdistance and RNApdist values, and the highest F1-scores among those achieved by the other algorithms. This highlights its precision, while the narrower spread of the violin plot reflects reduced variability in the results. However, the longer upper whisker compared to RNAinverse and Meta-LEARNA, which has the shortest, suggests that while DesiRNA generally delivers strong performance, it occasionally produces predictions with lower accuracy.

Meta-LEARNA achieves good and stable performance across all evaluation metrics. It is consistently high in F1-score, with low RNApdist and RNAdistance values, meaning that the predicted structures are well-aligned to the target. The distribution of its results is compact, with quite a narrow interquartile range and the shortest upper whisker, reflecting both high accuracy and low variability.

However, it is important to note that in the presented results, Meta-LEARNA produced outputs for only 2748 out of 4921 samples, which is significantly fewer than the other evaluated algorithms. In this regard, Meta-LEARNA performs the worst in terms of coverage. Since the metrics are reported only on the common instances addressed by all tools, this limitation is not reflected in the primary performance scores. Nonetheless, the impact of incomplete coverage becomes evident in the heatmap-based analysis of the one-sided Wilcoxon signed-rank test p-values, where differences in the number of valid predictions are taken into account.

RNAinverse and DSS-Opt exhibit a cluster of low RNAdistance values, which implies accurate RNA design predictions. Additionally, their interquartile range is narrow, though slightly wider than that of DesiRNA, reflecting consistent performance across the evaluated algorithms. The relatively compact distribution further suggests that most predictions are close to the median, with fewer extreme outliers.

INFO-RNA displays a wider distribution than DesiRNA, Meta-LEARNA and RNAinverse, indicating greater variability in its predictions. It shows moderate upper whiskers, reflecting occasional high values, but not as high as RNAsfbinv and RNARedPrint. Although its RNApdist values are very close to those of DSS-Opt, the higher RNAdistance values suggest that INFO-RNA is less consistent in accurately predicting RNA sequences compared to other algorithms. Furthermore, INFO-RNA performs worse in predicting 3-way junction motifs compared to DSS-Opt and RNAinverse.

RNAsfbinv and RNARedPrint display similar distributions and interquartile ranges, indicating comparable variability in their predictions. However, RNARedPrint has a lower median, which is comparable to that of other algorithms, except in cases involving 3-way junction motifs, where it performs poorly. Beyond 3-way junctions, RNARedPrint, INFO-RNA, and RNAsfbinv show similar behavior and exhibit slightly reduced performance, in contrast to the relatively robust results achieved by the remaining algorithms.

**Fig. 5:**
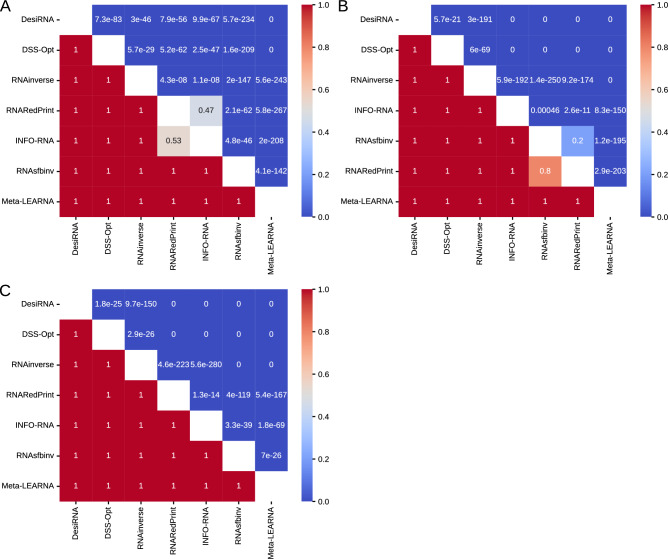
The heatmaps of one-sided Wilcoxon signed-rank tests for RNAsolo dataset. (**A**) RNApdist metric. (**B**) RNAdistance metric. (**C**) F1-score.

Analysis of the heatmaps of the one-sided Wilcoxon signed-rank test p-values (Fig. 5) reveals a consistent pattern. Among the three metrics analyzed, DesiRNA, DSS-Opt, and RNAinverse emerged as the top performers in that order. Meanwhile, Meta-LEARNA displayed the weakest performance. The middle tier includes RNARedPrint, INFO-RNA, and RNAsfbinv, with their rankings varying depending on the specific metric used. While the F1-score provides a clear distinction among these methods, this clarity is not observed with the RNApdist and RNAdistance metrics. According to the RNApdist metric, RNARedPrint and INFO-RNA do not significantly outperform each other. Similarly, the RNAdistance metric shows no statistically significant difference in performance between RNAsfbinv and RNARedPrint.

**Fig. 6:**
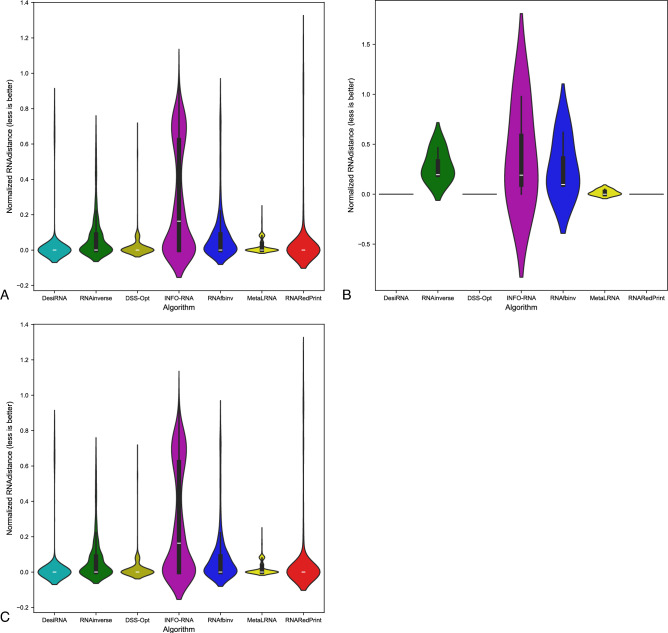
RNA design tools’ performance on Rfam dataset, illustrated by the example of the glutamine riboswitch (RFAM id: RF01739), using normalized RNAdistance for benchmarking. (**A**) The entire set. (**B**) The subset that contains internal loops. (**C**) The subset that contains 3-way junctions. The Meta-LEARNA algorithm is labeled as MetaLRNA in the figure for short.

For the dataset extracted from RNAsolo, DesiRNA stands out as the most reliable tool for RNA structure prediction, consistently achieving the lowest RNApdist and normalized RNAdistance values, along with the highest F1-scores across all datasets. RNAinverse and DSS-Opt exhibit good effectiveness, whereas INFO-RNA, RNAsfbinv and RNARedPrint show moderate performance. Meta-LEARNA, on the other hand, displayed the weakest performance among the evaluated tools.

#### Benchmarking test case using a dataset of loop motifs derived from the Rfam database

Here, similar to the previous example, we divided the analyzed datasets derived from the Rfam database (Rfam IDs: RF01739, RF02681, and RF00009) into the following subsets: internal loops and other higher-cardinality junctions. From these, we further distinguished two additional subsets: 3-way junctions and 4-way junctions. We utilized these datasets to evaluate and compare the performance of several RNA design tools: RNAinverse, INFO-RNA, DSS-Opt, RNAsfbinv, RNARedPrint, Meta-LEARNA, and DesiRNA. The analysis was conducted on instances common to all tools, with their performance assessed using three metrics: RNAdistance, RNApdist and F1-score.

*Benchmarking test case using a loop motifs dataset derived from the Rfam database, illustrated by the example of the glutamine riboswitch (RFAM id: RF01739)*

As the first example of a dataset derived from the Rfam database, we selected the RF01739 (glutamine riboswitch) family due to its inclusion of a significant and conserved 3-way junction^72–74^. It plays a central biological role, serving as the core structural element that undergoes ligand-induced rigidification upon L-glutamine binding, thereby mediating a conformational switch essential for metabolite sensing and gene regulation^73,79^. Furthermore, this riboswitch is unique in the following respects: most notably, ligand binding stabilizes the aptamer in an open conformation, in contrast to the closed state typically favored in other riboswitch classes^80^. This alignment comprises over 1,700 sequences and encompasses more than 2,200 loops.

**Fig. 7:**
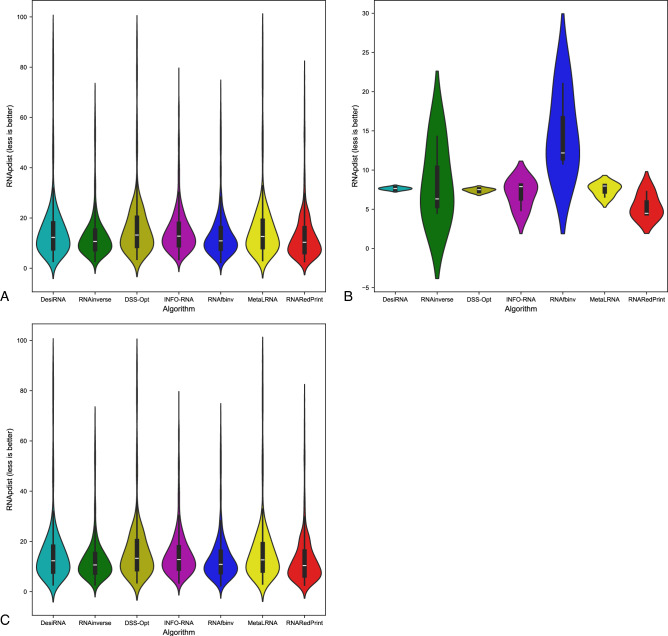
RNA design tools’ performance on Rfam dataset, illustrated by the example of the glutamine riboswitch (RFAM id: RF01739), using RNApdist for benchmarking. (**A**) The entire set. (**B**) The subset that contains internal loops. (**C**) The subset that contains 3-way junctions. The Meta-LEARNA algorithm is labeled as MetaLRNA in the figure for short.

**Fig. 8:**
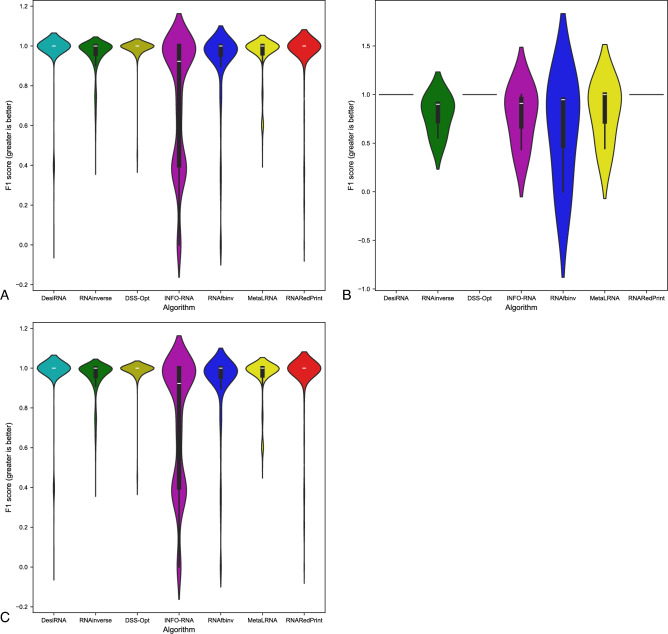
RNA design tools’ performance on Rfam dataset, illustrated by the example of the glutamine riboswitch (RFAM id: RF01739), using F1-score for benchmarking. (**A**) The entire set. (**B**) The subset that contains internal loops. (**C**) The subset that contains 3-way junctions. The Meta-LEARNA algorithm is labeled as MetaLRNA in the figure for short.

The results are shown in Table 8 and Figs. 6, 7 and 8. Upon analysis, it is clear that only two subsets are considered: those involving internal loops and 3-way junctions. This is due to the absence of higher-order branching junctions in the analyzed RNA family.

**Table 8:** RNA design benchmark results for the Rfam dataset, illustrated by the example of the glutamine riboswitch (RFAM id: RF01739), divided by motif type (best values in bold).

RNA design algorithm	Average computing time (s)	Normalized RNAdistance	RNApdist	F1-score
Results for 931 instances successfully solved by each algorithm				
RNAinverse	0.52	0.07	13.02	0.95
RNAsfbinv	3.89	0.09	13.48	0.92
INFO-RNA	**0.27**	0.28	15.06	0.73
RNARedPrint	7.78	0.06	**12.98**	0.96
DSS-Opt	1.05	**0.02**	16.75	**0.99**
DesiRNA	411.56	0.03	15.57	0.97
Meta-LEARNA	3.13	**0.02**	16.09	0.96
Results for 3 instances of internal loop motifs successfully solved by each algorithm				
RNAinverse	0.14	0.28	8.36	0.79
RNAsfbinv	3.45	0.27	14.66	0.63
INFO-RNA	**0.03**	0.39	6.97	0.78
RNARedPrint	6.64	**0.00**	**5.40**	**1.00**
DSS-Opt	0.88	**0.00**	7.41	**1.00**
DesiRNA	379.02	**0.00**	7.64	**1.00**
Meta-LEARNA	3.37	0.02	7.51	0.81
Results for 928 instances of 3-way junction motifs successfully solved by each algorithm				
RNAinverse	0.52	0.07	13.02	0.95
RNAsfbinv	3.89	0.09	13.48	0.92
INFO-RNA	**0.27**	0.28	15.06	0.73
RNARedPrint	7.79	0.06	**13.01**	0.96
DSS-Opt	1.05	**0.02**	16.78	**0.99**
DesiRNA	411.67	0.03	15.60	0.97
Meta-LEARNA	3.13	**0.02**	16.12	0.96

Among the tools evaluated, RNAinverse demonstrates high accuracy and reliability in RNA sequence prediction, consistently achieving low RNAdistance and RNApdist values, along with a high F1-score. It consistently generates sequences with structures close to the target for all subsets, with the exception of the one containing internal loops. DesiRNA maintains very good performance, with a low median but a larger number of outliers than RNAinverse and Meta-LEARNA. It is robust across all datasets but shows occasional outliers. Meta-LEARNA emerges as a competitive method, combining consistently low error rates with high F1-scores across all motifs. Together with DSS-Opt, it achieves the best RNAdistance and F1-score for internal loops and maintains strong performance on 3-way junction motifs. RNAsfbinv also performs well, particularly in terms of RNAdistance and F1-score values. INFO-RNA shows moderate performance with greater variability in predictions. It is generally reliable, but less consistent than DesiRNA, Meta-LEARNA and RNAinverse. RNARedPrint demonstrates very good performance across all motif categories. It achieves one of the highest F1-scores and the lowest RNApdist, along with low RNAdistance. On the other hand, DSS-Opt shows a similar distribution to Meta-LEARNA and DesiRNA, with an upper whisker comparable in width to DesiRNA. This indicates that DSS-Opt demonstrates consistent performance and reliability in predicting RNA structures for most instances within the evaluated dataset.

As we were particularly interested in the 3-way junction motif in this example, we took a closer look at the performance of RNA design algorithms for the subset containing these motifs. For predicting 3-way junction motifs, DesiRNA, DSS-Opt, and Meta-LEARNA exhibited very similar distributions, reflecting high accuracy and consistency. Among these, Meta-LEARNA and DSS-Opt achieved the best results for the normalized RNAdistance metric, while RNARedPrint outperformed the others in RNApdist. All algorithms, except INFO-RNA, displayed relatively compact distributions with low median values. INFO-RNA, on the other hand, had a wide distribution and a noticeably higher median value, indicating more variability and less consistency in approximating the target structure.

**Fig. 9:**
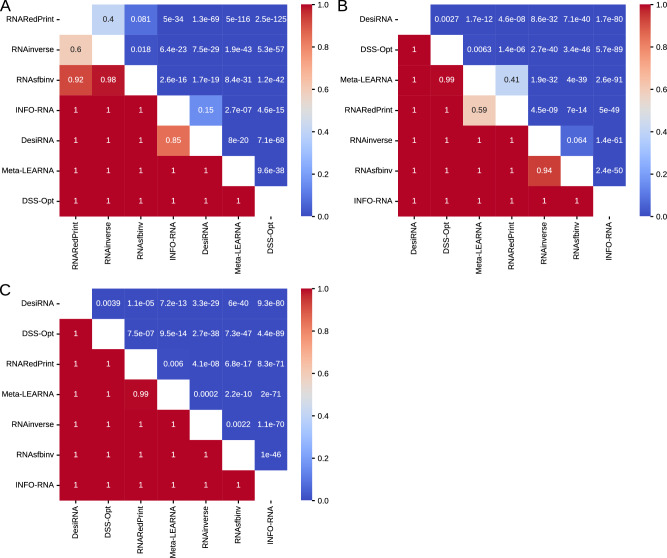
The heatmaps of one-sided Wilcoxon signed-rank tests for the glutamine riboswitch (RFAM id: RF01739) dataset. (**A**) RNApdist metric. (**B**) RNAdistance metric. (**C**) F1-score.

The heatmaps illustrating the p-values from one-sided Wilcoxon signed-rank tests (Fig. 9) reveal distinct performance patterns across the distance metrics. When evaluating sequence similarity using RNApdist, a clear distinction arises among the methods. RNARedPrint, RNAinverse, and RNAsfbinv generally perform better than the other four methods: DSS-Opt, Meta-LEARNA, DesiRNA, and INFO-RNA. Among the top three methods, the differences in performance are less pronounced or not consistently significant. Similarly, INFO-RNA and DesiRNA do not show significant differences in the middle tier.

Turning to structural comparison metrics, such as RNAdistance and F1-score, the performance landscape changes significantly, and both metrics yield similar conclusions. INFO-RNA consistently performs worse than all other methods, as indicated by p-values of 1.0 in its row and near-zero p-values in its column across both heat maps. In stark contrast to the RNApdist results, DSS-Opt and DesiRNA emerge as top performers in structural accuracy. They significantly outperform INFO-RNA, RNARedPrint, RNAinverse, and RNAsfbinv based on both RNAdistance and F1-score, as evidenced by numerous very small p-values in the rows for DSS-Opt and DesiRNA against these methods. Meta-LEARNA also performs structurally well, significantly better than INFO-RNA, RNAinverse, and RNAsfbinv. However, it does not consistently show a significant advantage over RNARedPrint and is generally outperformed by DSS-Opt and DesiRNA.

This analysis highlights that for the glutamine riboswitch target, DesiRNA excels under the structural metrics, while RNARedPrint and RNAinverse dominate when evaluated with RNApdist.

*Benchmarking test case using a loop motifs dataset derived from the Rfam database, illustrated by the example of the twister sister ribozyme (RFAM id: RF02681)*

**Fig. 10:**
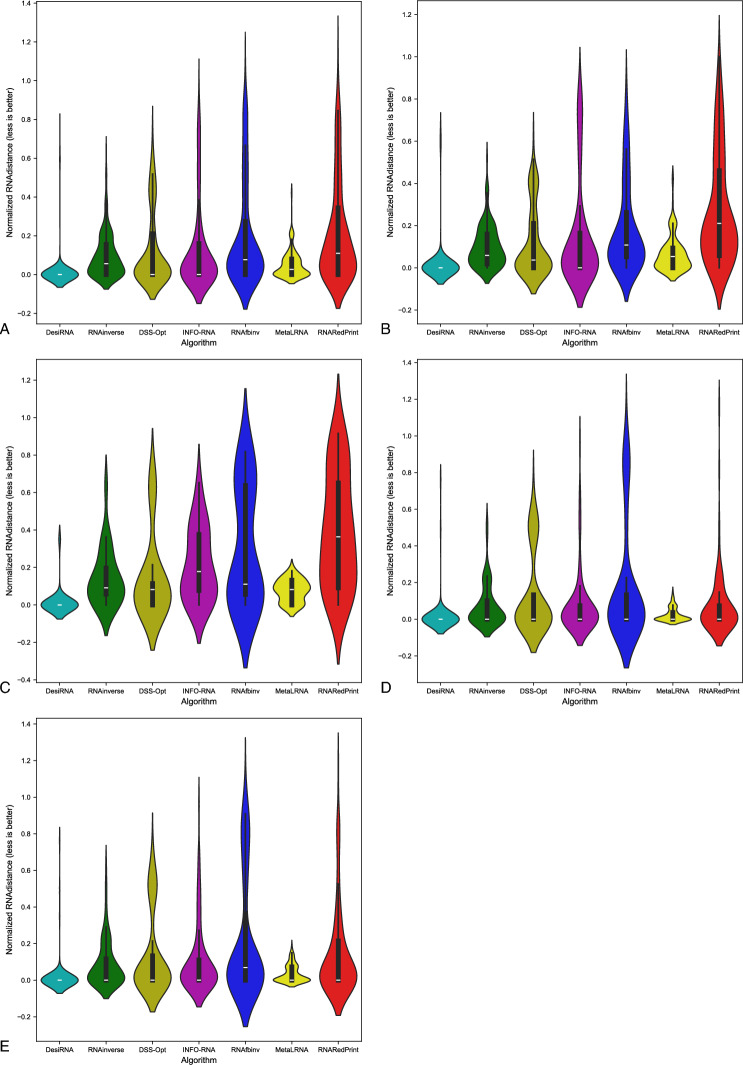
RNA design tools’ performance on Rfam dataset, illustrated by the example of the twister sister ribozyme (RFAM id: RF02681), using normalized RNAdistance for benchmarking. (**A**) The entire set. (**B**) The subset that contains internal loops. (**C**) The subset that contains 3-way junctions. (**D**) The subset that contains 4-way junctions. (**E**) The subset that contains other higher-cardinality junctions. The Meta-LEARNA algorithm is labeled as MetaLRNA in the figure for short.

**Fig. 11:**
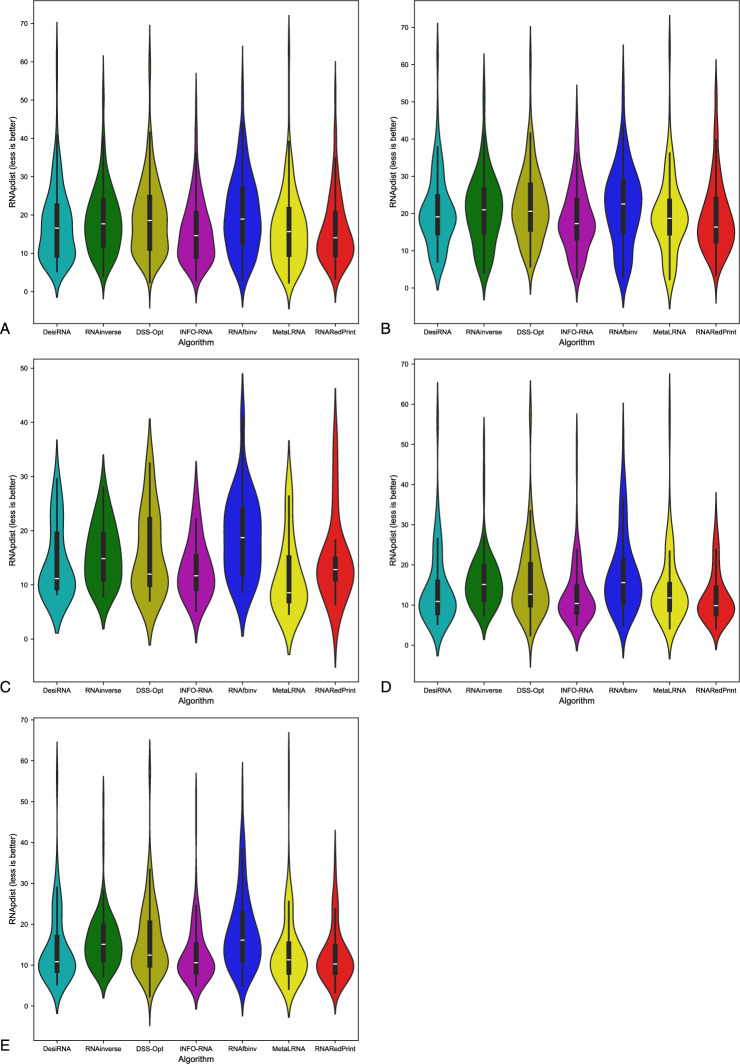
RNA design tools’ performance on Rfam dataset, illustrated by the example of the twister sister ribozyme (RFAM id: RF02681), using RNApdist for benchmarking. (**A**) The entire set. (**B**) The subset that contains internal loops. (**C**) The subset that contains 3-way junctions. (**D**) The subset that contains 4-way junctions. (**E**) The subset that contains other higher-cardinality junctions. The Meta-LEARNA algorithm is labeled as MetaLRNA in the figure for short.

**Fig. 12:**
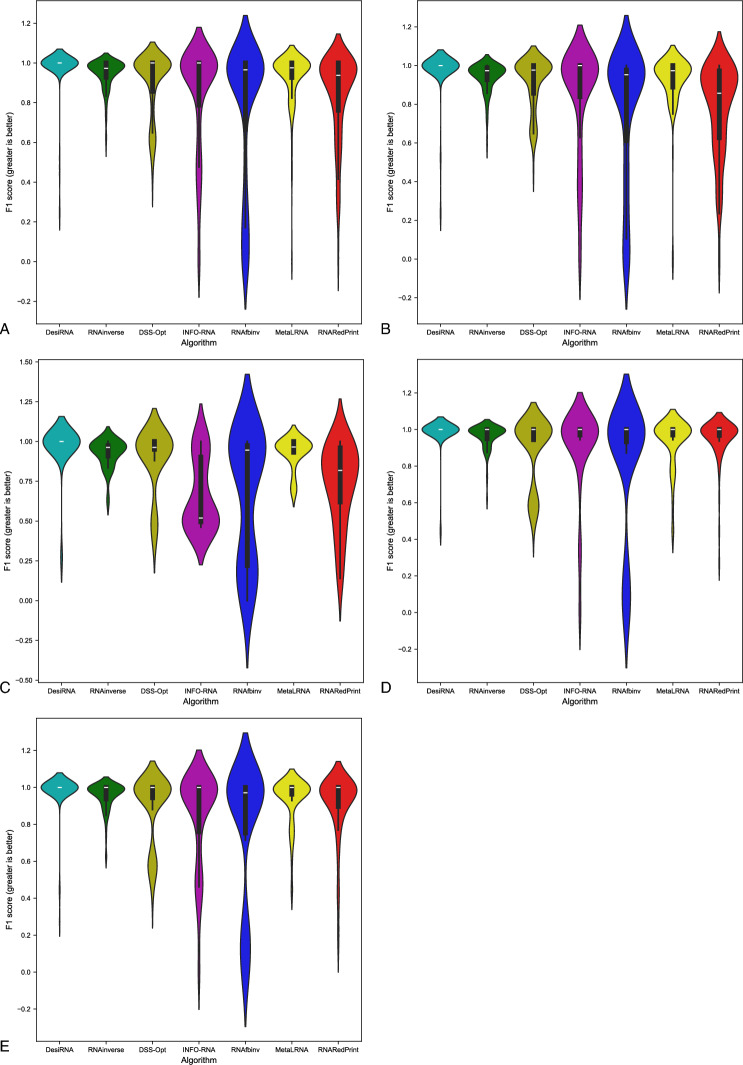
RNA design tools’ performance on Rfam dataset, illustrated by the example of the twister sister ribozyme (RFAM id: RF02681), using F1-score for benchmarking. (**A**) The entire set. (**B**) The subset that contains internal loops. (**C**) The subset that contains 3-way junctions. (**D**) The subset that contains 4-way junctions. (**E**) The subset that contains other higher-cardinality junctions. The Meta-LEARNA algorithm is labeled as MetaLRNA in the figure for short.

Another example of a dataset derived from the Rfam database is the RF02681 family (the twister sister ribozyme). This family is particularly intriguing due to the presence of the 4-way junction motif^75,76^, which offers valuable insights into the performance and capabilities of RNA design algorithms. It possesses well-defined and highly conserved secondary structure, which underlies its catalytic activity. Furthermore, the ribozyme’s cleavage activity is strongly dependent on specific secondary structure motifs, including stem–loop arrangements and the central 4-way junction, which are essential for correctly positioning catalytic residues. The junction forms a stabilizing network that organizes distant structural elements and supports the formation of an active catalytic core^76,81,82^. The results for this dataset are shown in Table 9 and Figs. 10, 11 and 12.

**Table 9:** RNA design benchmark results for the Rfam dataset, illustrated by the example of the twister sister ribozyme (RFAM id: RF02681), divided by motif type (best values in bold).

RNA design algorithm	Average computing time (s)	Normalized RNAdistance	RNApdist	F1-score
Results for 245 instances successfully solved by each algorithm				
RNAinverse	0.42	0.10	18.79	0.95
RNAsfbinv	7.22	0.20	20.37	0.77
INFO-RNA	**0.13**	0.13	**15.98**	0.84
RNARedPrint	9.12	0.11	16.30	0.83
DSS-Opt	1.16	0.13	19.52	0.90
DesiRNA	442.05	**0.02**	17.80	**0.98**
Meta-LEARNA	3.77	0.05	16.88	0.93
Results for 137 instances of internal loop motifs successfully solved by each algorithm				
RNAinverse	0.59	0.10	21.04	0.94
RNAsfbinv	9.73	0.19	22.11	0.76
INFO-RNA	**0.22**	0.16	**18.50**	0.84
RNARedPrint	9.16	0.28	19.29	0.78
DSS-Opt	1.24	0.12	22.25	0.91
DesiRNA	442.39	**0.02**	20.67	**0.98**
Meta-LEARNA	3.91	0.05	19.50	0.93
Results for 135 instances of higher-cardinality junction motifs successfully solved by each algorithm				
RNAinverse	0.21	0.09	16.03	0.95
RNAsfbinv	4.12	0.22	18.23	0.77
INFO-RNA	**0.03**	0.11	12.89	0.85
RNARedPrint	9.07	0.15	**12.64**	0.90
DSS-Opt	1.05	0.14	16.17	0.89
DesiRNA	441.63	**0.01**	14.28	**0.99**
Meta-LEARNA	3.60	0.03	13.67	0.93
Results for 24 instances of 3-way junction motifs successfully solved by each algorithm				
RNAinverse	0.17	0.15	15.72	0.93
RNAsfbinv	4.33	0.31	19.19	0.66
INFO-RNA	**0.03**	0.21	13.05	0.67
RNARedPrint	8.79	0.39	14.89	0.73
DSS-Opt	1.03	0.15	16.17	0.89
DesiRNA	427.37	**0.01**	14.27	**0.97**
Meta-LEARNA	5.08	0.08	**11.70**	0.93
Results for 86 instances of 4-way junction motifs successfully solved by each algorithm				
RNAinverse	0.22	0.07	16.12	0.96
RNAsfbinv	4.07	0.19	17.96	0.80
INFO-RNA	**0.03**	0.08	12.85	0.90
RNARedPrint	9.15	0.08	**12.02**	0.95
DSS-Opt	1.06	0.14	16.17	0.89
DesiRNA	445.61	**0.01**	14.29	**0.99**
Meta-LEARNA	3.19	0.02	14.22	0.93

For this dataset, DesiRNA stands out among the evaluated tools, exhibiting the highest accuracy and reliability in RNA sequence prediction, with consistently low RNAdistance and RNApdist values and high F1-scores. It reliably produces sequences with structures that closely match the target across all subsets. RNAinverse and Meta-LEARNA follows closely, also achieving low RNAdistance values, along with high F1-scores and showing reliable performance with minimal variability. DSS-Opt exhibits solid results, with moderate accuracy and consistency, performing slightly below DesiRNA and RNAinverse. INFO-RNA achieves the best values for the RNApdist metric, but its performance for normalized RNAdistance is only average, particularly for 3-way junctions. While generally reliable, it exhibits greater variability in predictions. RNAsfbinv and RNARedPrint achieve average performance, characterized by broader distributions and occasional inaccuracies. RNARedPrint, in particular, shows higher variability and elevated median values.

Focusing specifically on the 4-way junction motif, DesiRNA emerges as the best-performing tool, achieving the lowest normalized RNAdistance while demonstrating both accuracy and consistency. RNAinverse also performs well, with low median values, though it shows slightly greater variability compared to DesiRNA. DSS-Opt achieves moderate success, producing results similar to RNAinverse but with a broader distribution, indicating some inconsistencies. RNARedPrint produces results comparable to RNAinverse and INFO-RNA in terms of normalized RNAdistance while achieving the advantage of lower RNApdist values. However, its longer upper whisker suggests greater variability in its predictions, indicating occasional inconsistencies. RNAsfbinv performs worse, with broader distributions and with wider interquartile ranges, reflecting significant challenges in approximating the target structures. These findings highlight the challenges posed by higher-order junctions and underscore the need for further advances in RNA design tools.

**Fig. 13:**
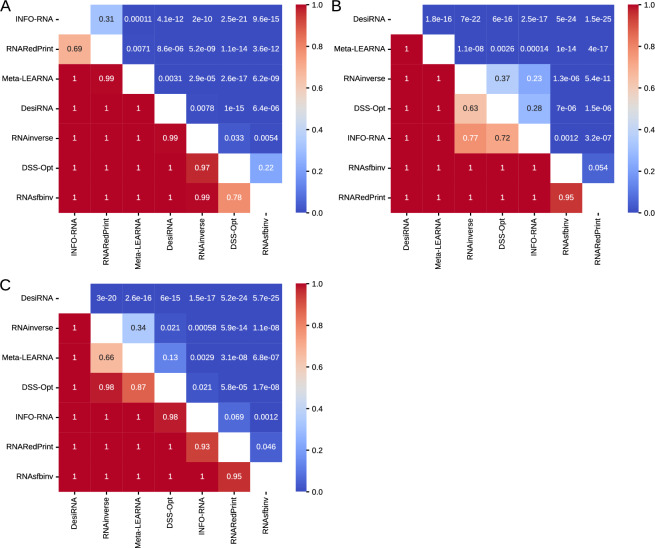
The heatmaps of one-sided Wilcoxon signed-rank tests for the twister sister ribozyme (RFAM id: RF02681) dataset. (**A**) RNApdist metric. (**B**) RNAdistance metric. (**C**) F1-score.

An analysis of one-sided Wilcoxon signed-rank tests (Fig. 13) for the Twister Sister ribozyme design results shows distinct performance patterns across the distance metrics. When RNApdist is used as the quality measure, INFO-RNA, RNARedPrint, and Meta-LEARNA tend to outperform their competitors in head-to-head comparisons. While DesiRNA convincingly beats DSS-Opt, RNAinverse, and RNAsfbinv, the three leading methods outperform it. DSS-Opt and RNAsfbinv are mutually not outperforming each other, placing them at the bottom of the rankings for this metric.

The ranking shifts when the secondary-structure edit distance (RNAdistance) is analyzed. DesiRNA dominates, achieving extremely low p-values against all six competitors and never losing a single matchup. Meta-LEARNA holds a strong second position. The middle tier is occupied by RNAinverse, DSS-Opt, and INFO-RNA, which do not show significant differences between each other. RNAsfbinv and RNARedPrint take the last two positions, with the former showing better results but on the verge of statistical significance.

The F1 score, which combines sensitivity and precision of the predicted secondary structures, again shows that DesiRNA leads the chart with minimal p-values against all other algorithms. Meta-LEARNA and RNAinverse form the next tier, achieving significant wins over INFO-RNA, RNARedPrint, RNAsfbinv, and sometimes over DSS-Opt. RNAsfbinv consistently trails the field, failing to defeat any rival at the 5% significance level.

*Benchmarking test case using a loop motifs dataset derived from the Rfam database, illustrated by the example of nuclear ribonuclease P (RNase P) (RFAM id: RF00009)*

The final example of a dataset derived from the Rfam database that we selected is the RF00009 family (nuclear ribonuclease P). This family is distinguished by its highly complex secondary structure, featuring a variety of motifs such as internal loops, 3-way junctions, and higher-cardinality junctions^77,78^. Nuclear RNase P is a highly conserved and ubiquitous endoribonuclease found in all domains of life, bacteria, archaea, and eukarya, as well as in organelles such as mitochondria and chloroplasts. Its primary and best-characterized function is the processing of precursor tRNAs, specifically through cleavage of the 5’ leader sequence to produce mature tRNAs. Although bacterial RNase P RNA is catalytically active on its own, functioning as a ribozyme, the eukaryotic version operates as a large ribonucleoprotein complex where the RNA component is catalytically inactive in isolation but remains essential for holoenzyme function. Despite considerable evolutionary divergence, particularly in sequence among eukaryotes, all RNase P RNAs share a conserved catalytic core, including helices P1, P2, P3, P4, and P10/11^77,78,83^. This evolutionary conservation of secondary structure motifs, combined with the functional complexity of eukaryotic RNase P, highlights its relevance for evaluating the performance of secondary structure-based RNA design algorithms.

**Fig. 14:**
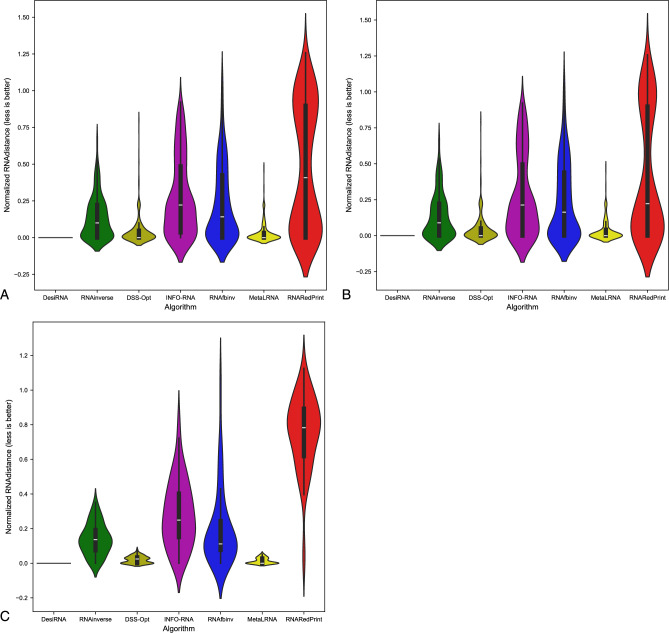
RNA design tools’ performance on Rfam dataset, illustrated by the example of nuclear ribonuclease P (RNase P) (RFAM id: RF00009), using normalized RNAdistance for benchmarking. (**A**) The entire set. (**B**) The subset that contains internal loops. (**C**) The subset that contains 4-way junctions. The Meta-LEARNA algorithm is labeled as MetaLRNA in the figure for short.

**Fig. 15:**
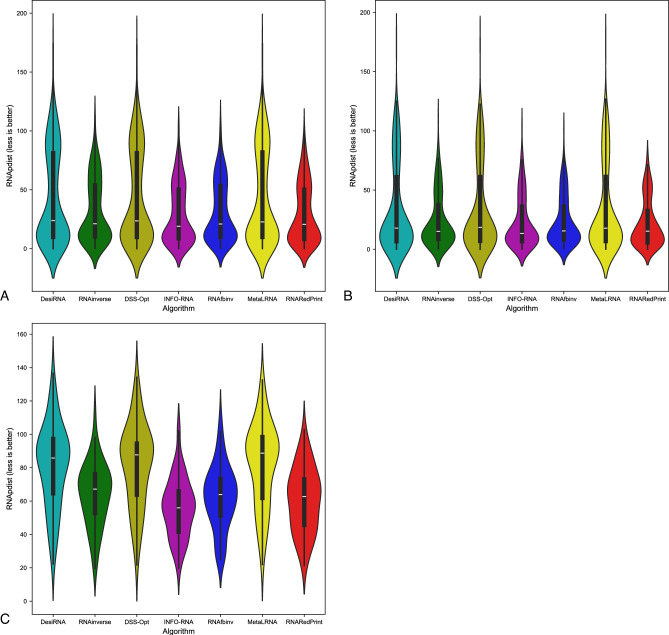
RNA design tools’ performance on Rfam dataset, illustrated by the example of nuclear ribonuclease P (RNase P) (RFAM id: RF00009), using RNApdist for benchmarking. (**A**) The entire set. (**B**) The subset that contains internal loops. (**C**) The subset that contains 4-way junctions. The Meta-LEARNA algorithm is labeled as MetaLRNA in the figure for short.

**Fig. 16:**
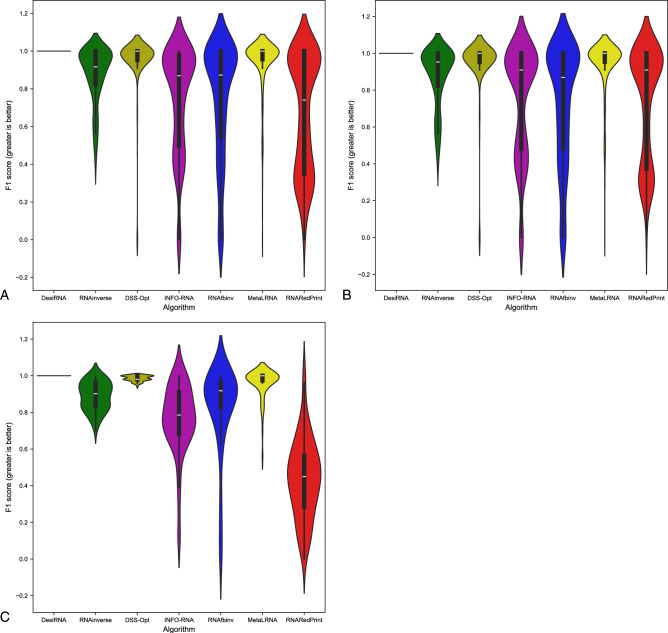
RNA design tools’ performance on Rfam dataset, illustrated by the example of nuclear ribonuclease P (RNase P) (RFAM id: RF00009), using F1-score for benchmarking. (**A**) The entire set. (**B**) The subset that contains internal loops. (**C**) The subset that contains 4-way junctions. The Meta-LEARNA algorithm is labeled as MetaLRNA in the figure for short.

The results are provided in Table 10 and Figs. 14, 15 and 16. As observed, the results include only subsets containing internal loops and 4-way junctions, with no data available for 3-way junctions. This absence is due to RNAsfbinv’s inability to generate sequences for structures containing 3-way junction motifs. As mentioned previously, given the varying accuracy levels of different algorithms across sequences of different lengths and the significant number of outliers produced by some tools, the analysis was limited to instances successfully handled by all approaches to ensure comparability.

**Table 10:** RNA design benchmark results for the Rfam dataset, illustrated by the example of nuclear ribonuclease P (RNase P) (RFAM id: RF00009), divided by motif type (best values in bold).

RNA design algorithm	Average computing time (s)	Normalized RNAdistance	RNApdist	F1-score
Results for 307 instances successfully solved by each algorithm				
RNAinverse	4.01	0.10	31.95	0.88
RNAsfbinv	15.86	0.14	31.01	0.74
INFO-RNA	3.52	0.22	**28.90**	0.74
RNARedPrint	10.12	0.41	29.39	0.67
DSS-Opt	**1.30**	**0.00**	43.48	0.96
DesiRNA	431.39	**0.00**	43.36	**1.00**
Meta-LEARNA	3.69	**0.00**	43.57	0.94
Results for 247 instances of internal loop motifs successfully solved by each algorithm				
RNAinverse	1.98	0.09	24.18	0.87
RNAsfbinv	8.08	0.16	23.39	0.72
INFO-RNA	1.97	0.21	22.25	0.73
RNARedPrint	9.44	0.22	**21.81**	0.73
DSS-Opt	**1.02**	**0.00**	34.17	0.95
DesiRNA	418.78	**0.00**	34.15	**1.00**
Meta-LEARNA	3.66	**0.00**	34.23	0.94
Results for 60 instances of 4-way junction motifs successfully solved by each algorithm				
RNAinverse	12.40	0.14	63.96	0.89
RNAsfbinv	47.89	0.11	62.36	0.81
INFO-RNA	9.91	0.25	**56.29**	0.76
RNARedPrint	12.94	0.78	60.62	0.44
DSS-Opt	**2.45**	0.02	81.83	0.99
DesiRNA	483.30	**0.00**	81.27	**1.00**
Meta-LEARNA	3.83	**0.00**	82.03	0.96

DesiRNA consistently demonstrates the best performance across all subsets when considering normalized RNAdistance and F1-score, achieving the lowest median values and a narrow distribution that reflects high consistency and reliability. However, when focusing on RNApdist, DesiRNA shows slightly higher median values compared to other algorithms, particularly for 4-way junctions. Its violin plots also reveal occasional outliers, as indicated by a slightly longer upper whisker. Nevertheless, it performs exceptionally well in producing sequences closely matching target structures and maintains competitive performance, especially in terms of overall stability and accuracy for complex motifs. DSS-Opt presents similar results, delivering strong performance with low median values and compact distributions, though slightly wider than those of DesiRNA, indicating consistent and reliable predictions.

When considering RNApdist as the evaluation metric, INFO-RNA, RNAsfbinv, RNAinverse and RNARedPrint emerge as the top performers. In particular, the median values of the RNApdist are similar across all algorithms, except for 4-way junctions, where DesiRNA and DSS-Opt show slightly elevated values. The consistency of INFO-RNA and RNARedPrint in RNApdist reinforces its ability to capture the properties of the ensemble. However, they do not match the precision of DesiRNA and DSS-Opt in normalized RNA distance predictions.

Meta-LEARNA demonstrates strong overall performance across the evaluated datasets, achieving low median RNAdistance and RNApdist values along with consistently high F1-scores, placing it among the top-performing algorithms. Its predictions exhibit low variability, as seen in narrow interquartile ranges and minimal outliers across different motif types.

RNAinverse performs well, achieving low RNAdistance and RNApdist values with a tight interquartile range. Its narrow violin plot highlights its consistency and low variability in predictions. The low median values in all subsets emphasize its reliability and accuracy, although it falls slightly behind DesiRNA and DSS-Opt in overall performance.

RNAsfbinv demonstrates variable performance, characterized by broader violin plots and higher upper whiskers for RNAdistance, with predictions showing less consistency, as reflected in the wide spread of results.

**Fig. 17:**
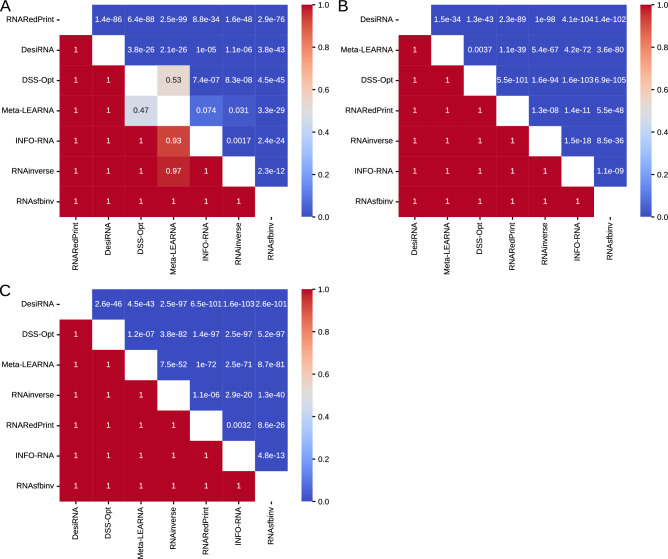
The heatmaps of one-sided Wilcoxon signed-rank tests for the nuclear ribonuclease P (RNase P) (RFAM id: RF00009) dataset. (**A**) RNApdist metric. (**B**) RNAdistance metric. (**C**) F1-score.

Based on one-sided Wilcoxon signed-rank test p-values (Fig. 17) for sequence similarity (measured using RNApdist) on the RF00009 benchmark, RNARedPrint significantly outperforms all other methods, yielding p-values well below conventional significance thresholds (often $<<$ 1e–30). This suggests that RNARedPrint generates sequences significantly closer to the ground truth than its competitors. DesiRNA also performs well, demonstrating significant improvements over the remaining methods (RNAinverse, RNAsfbinv, DSS-Opt, Meta-LEARNA, and INFO-RNA). Conversely, RNAinverse and RNAsfbinv perform poorly. DSS-Opt, Meta-LEARNA, and INFO-RNA form a middle tier, with no statistically significant performance differences observed between DSS-Opt and Meta-LEARNA, nor between Meta-LEARNA and INFO-RNA.

Regarding structural accuracy, the RNAdistance and F1-score metrics results show both consistencies and differences. According to both metrics, DesiRNA performs best, while RNAsfbinv performs worst. Meta-LEARNA and DSS-Opt rank second and third, with their specific positions varying depending on the metric. Similarly, the middle tier, comprising RNARedPrint, RNAinverse, and INFO-RNA, also shows varying ranks depending on the metric.

Evaluating RNA inverse folding methods reveals performance differences depending on the metric. RNARedPrint excels significantly in sequence similarity, producing sequences much closer to the ground truth than competitors, followed by DesiRNA. However, for structural accuracy (measured by RNAdistance and F1-score), DesiRNA ranks best, while RNARedPrint falls into the middle tier. RNAsfbinv consistently performs poorly across both sequence similarity and structural accuracy metrics. Other methods like DSS-Opt, Meta-LEARNA, and INFO-RNA occupy intermediate positions, with their relative rankings sometimes shifting between sequence similarity and structural accuracy assessments.

These findings underscore the variability in performance evaluation of RNA design methods depending on the distance metric used, with DesiRNA and RNARedPrint excelling under different criteria.

## Discussion

In the rapidly evolving field of RNA bioinformatics, the growing application of machine learning has increased the demand for high-quality, comprehensive data resources. Our newly developed dataset of multiloop motifs in RNA structures addresses this critical need by merging information from experimentally solved 3D structures with the extensive sequence repository of Rfam, a well-known database of RNA families and their sequence alignments.

This resource contains over 320,000 entries of authentic RNA motifs, including internal loops, three-way junctions, four-way junctions, and higher cardinality junctions. Importantly, these motifs are derived from experimentally verified data rather than synthetic constructs, providing researchers with reliable reference materials. The dataset allows each motif to be described in isolation or within its structural context, offering flexibility for diverse analytical approaches.

We systematically evaluated several inverse folding algorithms using multiple comparison metrics to demonstrate the dataset’s utility. Our analyses revealed distinct patterns across different RNA families. DesiRNA exhibited exceptional performance, ranking among the top in all normalized RNAdistance and F1-score evaluations and performing strongly in most RNApdist tests. However, it showed moderate results for the glutamine riboswitch and was surpassed by INFO-RNA in RNApdist evaluations for the twister sister ribozyme. DSS-Opt often placed second in RNAdistance measurements while showing variable performance with RNApdist. Methods such as RNAinverse, Meta-LEARN, INFO-RNA and RNARedPrint generally maintained middle positions in our rankings, occasionally excelling in specific scenarios—RNARedPrint and RNAinverse led for the glutamine riboswitch using RNApdist, while INFO-RNA ranked first for the twister sister ribozyme with the same metric. Similarly, RNAsfbinv typically underperformed except for the glutamine riboswitch, which ranked second using RNApdist.

The most notable performance variation was observed for the nuclear RNase P family, where the evaluation of RNA design methods strongly depended on the chosen distance metric, resulting in shifts in algorithm rankings. These results highlight the importance of employing multiple evaluation metrics when assessing algorithm performance across diverse RNA families. Furthermore, the varying performance of different tools highlights that their effectiveness is context-dependent and significantly influenced by the specific characteristics of the target RNA family. This suggests that the suitability of a particular inverse folding method may vary based on the target, indicating that no single tool is universally optimal across all biological contexts. This emphasizes the data-sensitive nature of current inverse folding approaches.

The observed variation in performance across different RNA families can be attributed to several factors. First, the imbalance in the Rfam Database plays a critical role, as the database is highly heterogeneous. Some families contain large seed alignments, resulting in strong covariance signals and high confidence in their consensus secondary structures, which likely enhances the performance of inverse folding tools. In contrast, families with fewer sequences provide less support, leading to greater uncertainty in the consensus structure and potentially affecting prediction accuracy.

Second, the complexity of RNA families significantly contributes to this variation. The structural and functional diversity of RNA families influences performance. For example, some non-coding RNAs have intricate tertiary structures essential for their functions, presenting complex challenges for sequence design. Other RNAs, such as those that serve dual regulatory and transcriptional roles, may encounter evolutionary constraints that limit their structural complexity.

Third, variation in sequence length and features is inherent to biological data. RNA families in Rfam vary widely in sequence length, ranging from short RNAs to multi-kilobase rRNAs, among other characteristics. While this diversity is beneficial for accurately representing biological reality, inverse folding methods will demonstrate varying degrees of success depending on the specific target.

Beyond algorithm benchmarking, we demonstrated how our dataset could be used to train machine-learning models for RNA family classification tasks. This application illustrates the dataset’s potential to support various computational approaches in RNA bioinformatics, particularly deep learning methods that require large volumes of high-quality training data.

These classification experiments explicitly support the “and beyond” aspect of our title. While our primary motivation was establishing a challenging benchmark for inverse folding, the dataset’s extensive breadth and depth make it a valuable resource for various other tasks. For instance, the comprehensive information on diverse loop and junction types (see Tables 1 and 2) makes it well-suited for developing methods to predict n-way junction families^84,85^ or for addressing other structure-related prediction challenges.

We anticipate that this comprehensive, experimentally backed resource will become a cornerstone for the broader research community, enabling scientists to refine RNA design algorithms and enhance machine learning pipelines. By providing this rich and diverse dataset, we aim to accelerate progress in RNA bioinformatics and facilitate groundbreaking innovations in this vital field of study.

## Methods

### Data sources

RNAsolo^64^ is a self-updating database for RNA 3D structures, curated from the Protein Data Bank (PDB). By stripping away non-RNA chains and organizing the remaining structures into equivalence classes, RNAsolo simplifies bioinformatics research. It offers seamless downloads of various data subsets—whether clustered by resolution, source, or format. Updated every Thursday, RNAsolo guarantees to always have access to the most current data. As of June 20, 2024, it hosts 15,049 RNA structures, organized into 3,356 equivalence classes, each exemplified by a cluster representative. RNAsolo’s user-friendly interface allows to search, sort, and download RNA structures effortlessly.

Rfam^65,86^ is an indispensable database that houses a vast array of non-coding RNA (ncRNA) families, each carefully defined by a seed multiple sequence alignment, a consensus secondary structure, and a covariance model. These elements are critical for annotating ncRNAs within nucleotide datasets, a task seamlessly executed using Infernal software^87^. Rfam and Infernal play a vital role in genome annotation pipelines for external data providers.

### Data preparation

Our dataset integrates data from Rfam 14.10 and RNAsolo 3.326. Rfam 14.10 provides consensus secondary (2D) structures for sequences within full alignments of each Rfam family, while RNAsolo 3.326 supplies non-redundant tertiary (3D) structures, which we annotate for their canonical 2D representations.

We utilized the seed alignments from Rfam 14.10 as the basis for data preparation. For each RNA family alignment file in STOCKHOLM format, we employed R-scape with the--Rfam and--cacofold parameters to incorporate all additional covarying pairs. Subsequently, we converted the structural data from Rfam and CaCoFold in STOCKHOLM format into the dot-bracket notation, ensuring the removal of pseudoknots to maintain compatibility with RNA design algorithms. This process yielded a secondary structure associated with each sequence in the seed alignment, which we stored in a separate file.

For the RNAsolo dataset, we employed the RNApdbee tool alongside seven integrated base pair analyzers: baRNAba, BPNET, FR3D, MAXIT, MC-Annotate, RNApolis Annotator, and RNAview. Notably, the widely used tool DSSR was not included in our list. Licensing considerations primarily drove our decision during the tool selection phase. The release of DSSR version 2.0 introduced licensing requirements that raised concerns about accessibility, including potential costs for academic research. Although the basic version is currently available free of charge for academic users, the previous uncertainty led us to prioritize tools with consistently free and permissive licenses to ensure the long-term reproducibility and accessibility of our methodology. Each input structure and analyzer setting produced a list of base pairs and an optimal dot-bracket notation. We categorized each input structure into “empty,” “gapped,” “multistrand,” or categories “1” through “7,” with the first three serving as filters to exclude sequences unsuitable for RNA design benchmarking due to the absence of base pairs, gaps in the 3D chain, or multiple chains. The remaining categories represent the level of agreement among base pair analyzers, where “7” indicates unanimous agreement and “3” indicates concordance among at most three analyzers.

We recognize that conflicts can arise among base-pair analyzers, particularly in classifying non-canonical interactions. To address this, we developed a unification protocol to systematically resolve these conflicts and effectively integrate information from all seven analyzers. Our protocol creates a consensus secondary structure by iteratively incorporating base pairs based on the level of agreement among the analyzers. We begin by including base pairs identified by all seven tools. Next, we add potential base pairs detected by six tools, those recognized by five, etc. Throughout this process, we ensure that each newly added base pair is compatible with the ones already included in the consensus structure and does not conflict with them. This hierarchical approach results in a final, well-formed, and conflict-free representation of the secondary structure, maximizing the use of consensus across various annotation tools and ensuring the accurate construction of the secondary structure core. Pseudoknots were removed, consistent with the Rfam data processing.

Our study primarily focuses on canonical base pairs, where we generally observe stronger agreement. Nevertheless, we implemented the unification procedure described above to address potential discrepancies. We found that minor differences, such as variations in how specific base pairs are identified, can affect the annotated lengths of helical stems. Fortunately, these variations typically do not influence the overall topology of the secondary structure or the types of junctions identified in the RNAsolo dataset, which is central to our research.

We did not perform a systematic evaluation to assess how using different subsets of annotation tools or adding others, such as DSSR, might impact the dataset’s quality within the context of our study. Instead, we relied on the curated structures provided by the RNAsolo dataset and our unification protocol. Given the strong agreement usually found for canonical pairs among various annotation tools, we expect that the final secondary structure assignments derived from RNAsolo will remain relatively robust, regardless of the specific combination of base pair annotators employed.

The decision to focus this benchmark on canonical secondary structures, specifically loop motifs like junctions, while excluding G-quadruplexes and pseudoknots for now, was made due to practical considerations related to data availability and compatibility with current computational methods. While G-quadruplexes are known to be prevalent in various genomes^88^, the availability of experimentally determined structural data needed for benchmarking is still relatively limited^89,90^. This scarcity makes constructing a comprehensive and reliable benchmark dataset for G-quadruplexes challenging.

The challenges regarding pseudoknots are twofold. First, many contemporary RNA inverse folding algorithms are primarily designed for pseudoknot-free secondary structures. This limitation would restrict the number of applicable methods in a benchmark that includes them. Second, one of our primary data sources, the Rfam database, mainly uses covariance models that generally do not capture pseudoknotted interactions. However, we acknowledge that efforts are underway within Rfam to incorporate these features more broadly.

Given these limitations, we have concentrated our initial benchmark efforts on canonical, pseudoknot-free secondary structures. This approach ensures broader applicability to existing methods and relies on more robustly curated structural data.

**Fig. 18:**
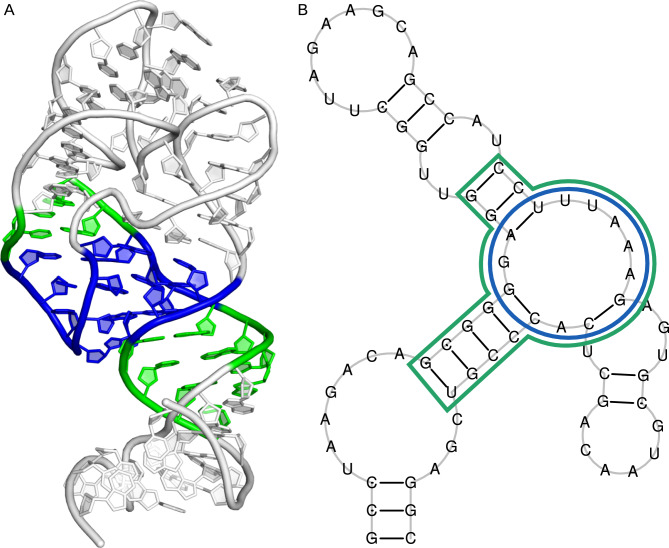
Structure of the base of ribosomal P stalk (PDB id: 5D8H, chain A). (**A**) 3D representation with the 3-way junction shown in blue and connecting stems shown in green. (**B**) 2D representation colored the same way.

Subsequently, we deconstructed each 2D structure into fundamental components: loops, stems, and single strands. Using the motif-extractor script from the RNApolis-py library, we automated this process. The script converts each dot-bracket notation into BPSEQ format and categorizes structural fragments based on predefined rules, such as recognizing adjacent base pairs as stems (Fig. 18).

To develop effective RNA design targets, we concentrated on loops, which are challenging to predict accurately. However, loops isolated from their structural context, such as connecting stems, are energetically unstable and unlikely to be independently predicted by RNA design algorithms. Therefore, for each identified loop motif, we generated three dataset instances: (1) the isolated loop fragment, (2) the loop fragment extended with its connecting stems and (3) the entire 2D structure containing the loop.

**Fig. 19:**
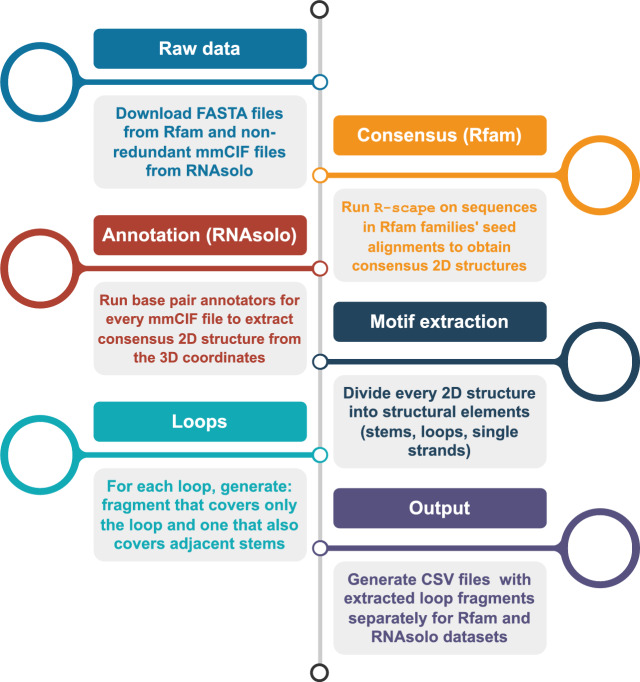
Data preparation pipeline: from Rfam and RNAsolo to extracted loop motifs.

The final step in our data preparation pipeline consolidates the results into a CSV file. Each row corresponds to a loop, with columns identifying the motif’s source and the sequence or dot-bracket encoded structure of the three instances mentioned above. The pipeline schematic is shown in Fig. 19.

### RNA design algorithms used for benchmarking and their evaluation

For the benchmarking experiments, we selected several open-source RNA design algorithms: RNAinverse, INFO-RNA, DSS-Opt, RNAsfbinv, RNARedPrint, Meta-LEARNA, and DesiRNA. All tools, except RNARedPrint, were executed using their default settings. By default, RNARedPrint uses a simple energy model and generates a uniform sample of sequences, as all feature weights (e.g., GC content and structural energies) are set to 1, effectively eliminating their influence. Therefore, we used the scripts provided by the authors, which implement a multidimensional Boltzmann sampling strategy on top of RNARedPrint. In addition, we employed a complementary script to directly identify sequences with high probabilities for the target structures. This script computes the minimum free energy (MFE) and ensemble energy (EE) for each sequence, followed by the calculation of individual target probabilities (Pi) and their sum (Psum). The final candidate was selected as the sequence with the highest Psum and the lowest MFE^52^. The code necessary to reproduce the analysis, including the commands for running these RNA design tools, is available at https://github.com/jbadura/rna_design/. Each script is designed to be run within a Docker container, with all required files accessible via the provided link.

To determine the secondary structure of each sequence generated during testing, we used RNAfold^15^. This methodology was chosen because not all programs provide sequence and structure in their results, necessitating a consistent method for evaluating prediction accuracy.

To compare the structural differences between the sequences designed by the examined tools and the original sequences, we utilized the RNAdistance^46^, RNApdist^15,46^ and F1-score^91^ methods. RNAdistance measures the structural difference between two RNA secondary structures by calculating the minimum number of edit operations needed to transform one structure into another using tree edit distance. This metric, part of the ViennaRNA package^15^, indicates more significant structural similarity with lower values. In contrast, RNApdist, also part of the ViennaRNA package, assesses distances between thermodynamic RNA secondary structure ensembles by calculating base pairing probability matrices. These matrices are condensed into vectors of pairing probabilities, which are then compared using a standard alignment algorithm. Additionally, the F1-score is used to evaluate the agreement between the predicted secondary structure of a generated sequence and the target structure. A higher F1-score reflects a closer match between the two structures.

Furthermore, to account for variability in RNA lengths and mitigate any indirect effects of sequence length on RNAdistance outcomes, we standardized the RNAdistance values. We divided each RNAdistance value by the corresponding RNA sequence length, allowing for a less biased comparison across different RNA sequences. Using violin plots, we evaluated and displayed the RNAdistance and RNApdist metrics, which reflect the efficiency of the various tools tested.

Using a one-sided Wilcoxon signed-rank test, we conducted statistical analyses to identify RNA design algorithms that significantly outperformed others. A critical aspect of this analysis was handling instances in which algorithms did not complete, whether due to timeouts or runtime exceptions. To fairly account for these failures in the pairwise comparisons, we assigned the worst possible value for the given metric to these instances. This approach ensures that a failing algorithm is guaranteed to lose the comparison for that specific case. Specifically, we assigned positive infinity ($+\infty$) to failures when evaluating RNApdist and RNAdistance and a value of 0 when assessing the F1-score. After completing these tests, we generated a heatmap of the p-values for each reported set of results and provided commentary on our findings.

All experiments were performed using a FormatServer THOR EHG21 system (Supermicro), equipped with two AMD EPYC 7543 32-core processors and 2 terabytes of RAM.

### Guidelines for evaluating machine learning models using the dataset

Evaluating machine learning (ML) models, especially for tasks like inverse folding or classification utilizing our dataset, necessitates a rigorous and comprehensive workflow to ensure reproducibility and fair comparisons. Although inverse folding can be performed using non-ML methods, it is essential to establish clear guidelines for evaluating ML models. Our manuscript outlines appropriate metrics, such as RNApdist for sequence-level comparisons and RNAdistance or F1-score for structure-level assessments (see Section *Evaluation and Comparison of RNA Design Algorithms’ Performance*). Additionally, we include distributional analysis and statistical testing (e.g., the Wilcoxon signed-rank test) to compare the performance of different methods.

A critical component of the ML evaluation workflow is the rigorous division of data into training and testing sets. We recommend the following best practices when using our dataset for ML applications:

- **Train/Test Split Ratio:** Employ standard ratios, such as 80% for training and 20% for testing, to provide sufficient data for model learning while retaining an independent set for evaluation.
- **Reproducible Shuffling:** Always shuffle the dataset before splitting, but use a fixed random seed to ensure that the split is reproducible for subsequent experiments or comparisons by others.
- **Stratified Splitting:** Given the potential imbalance in the distribution of features (e.g., different junction orders or RNA family types, which are notably imbalanced in the Rfam-derived portion of our dataset), use stratified splitting. This ensures that the proportion of key features is maintained across training and testing sets, preventing biased evaluation.
- **Group-Based Splitting:** To assess true generalization capabilities and prevent data leakage, consider group-based splitting, especially when dealing with related sequences. For instance, when using the Rfam portion of the dataset, ensure that all sequences belonging to the same Rfam family are assigned entirely to either the training or the testing set but not split across them. This step tests the model’s ability to generalize to unseen families or structural contexts.
- **K-Fold Cross-Validation:** For robust model training and hyperparameter tuning, apply k-fold cross-validation (e.g., 5-fold or 10-fold) exclusively on the training set. The final model performance should still be reported on the held-out test set.

Adhering to these guidelines will facilitate the development and reliable evaluation of ML-based models using the comprehensive datasets presented herein.

### Future directions: advanced machine-learning workflows enabled by the dataset

The diversity and precise annotations of multi-loop motifs contained in our benchmark open numerous avenues for state-of-the-art ML development that extend well beyond the baseline examples presented in this manuscript. Below, we outline ten concrete, non-mutually-exclusive research directions that the community can immediately pursue.

1. **Junction-centric graph neural networks (GNNs) for classification and family assignment.** Representing each junction as a heterogeneous graph whose nodes are residues and whose edges encode canonical as well as non-canonical interactions. Training equivariant GNNs to predict loop order, coaxial-stacking patterns, or Rfam family membership.
2. **Self-supervised pre-training of structural embeddings.** Applying contrastive or masked-node objectives to millions of unlabeled junction graphs to learn reusable embeddings that can be fine-tuned for downstream tasks such as ligand affinity prediction or mutational effect estimation.
3. **Conditional generative models for sequence design.** Developing diffusion or autoregressive models that generate RNA sequences conditioned on a fixed secondary-structure graph or on specific junction descriptors (e.g., loop cardinality, unpaired-length vector), enabling rapid in silico exploration of novel riboswitch scaffolds.
4. **Multi-task learning frameworks.** Jointly predicting (i) secondary structure, (ii) minimum free-energy difference to alternatives, and (iii) loop/junction category from a single network, thereby exploiting inductive transfer between thermodynamic and topological signals.
5. **Transfer learning from protein structure models.** Adapting large SE(3)-equivariant networks originally trained on proteins to RNA by fine-tuning on our dataset using geometric contrastive loss; early experiments suggest that backbone proximity statistics generalize surprisingly well across biopolymers.
6. **Few-shot meta-learning for rare high-order junctions.** Employing Model-Agnostic Meta-Learning (MAML) so that the network can quickly specialize to 7–12-way junctions, despite their scarcity, after seeing only a handful of examples.
7. **Active-learning loops coupled to folding simulators.** Using Bayesian uncertainty estimates from the classifier to query an external RNAfold engine for the most informative unlabeled motifs, iteratively enriching the training set where the model is least certain.
8. **Reinforcement-learning (RL) sequence editors.** Treating inverse folding as an RL environment where actions mutate nucleotides and rewards combine folding probability, ensemble diversity, and GC-content constraints; pretrained policies may then be fine-tuned on specific junction types.
9. **Structure-aware language models (”prompted RNA-LMs”).** Injecting linearized dot–bracket strings or tree encodings as prefixes (”prompts”) into large RNA language models so that token generation is implicitly guided by target structural contexts.
10. **Hybrid physics–ML surrogates.** Embedding differentiable nearest-neighbor or nearest-fragment energy terms inside neural architectures (e.g., via backprop-compatible McCaskill) to marry thermodynamic interpretability with data-driven accuracy.

By providing an unprecedented number of well-annotated internal loops and multi-branch junctions, our dataset supplies the balanced positive examples, rare-motif edge cases, and evaluation protocols required to benchmark each of the above ideas systematically. We expect that the next generation of RNA-specific GNNs, diffusion designers, and hybrid physics–ML methods will quickly adopt it as a standard development substrate.

## Data Availability

The datasets generated and/or analyzed during the current study are available in the Zenodo repository: https://zenodo.org/doi/10.5281/zenodo.12681122 All codes used for analyses presented in this paper are available in the GitHub repository: https://github.com/jbadura/rna_design
